# Computer-aided synthesis of dapsone-phytochemical conjugates against dapsone-resistant *Mycobacterium leprae*

**DOI:** 10.1038/s41598-020-63913-9

**Published:** 2020-04-22

**Authors:** Shasank S. Swain, Sudhir K. Paidesetty, Budheswar Dehury, Madhusmita Das, Sundeep C. Vedithi, Rabindra N. Padhy

**Affiliations:** 1 Central Research Laboratory, Institute of Medical Sciences and Sum Hospital, Siksha ‘O’ Anusandhan Deemed to be University, Kalinga Nagar, Bhubaneswar, 751003 Odisha India; 2 Division of Microbiology and NCDs, ICMR-Regional Medical Research Centre, Chandrasekharpur, Bhubaneswar, 751023 Odisha India; 3 Department of Pharmaceutical Chemistry, School of Pharmaceutical Sciences, Siksha ‘O’ Anusandhan Deemed to be University, Bhubaneswar, 751003 Odisha India; 4 ICMR-Regional Medical Research Centre, Chandrasekharpur, Bhubaneswar, 751023 Odisha India; 5 Department of Chemistry, Technical University of Denmark, DK-2800 Kongens Lyngby, Denmark; 6 Schieffelin Institute of Health-Research and Leprosy Centre, Karigiri, 632106 Tamil Nadu India; 7 Department of Biochemistry, University of Cambridge, Tennis Ct. Rd., CB2 1GA Cambridge, UK

**Keywords:** Computational biology and bioinformatics, Drug discovery, Drug discovery and development

## Abstract

Leprosy continues to be the belligerent public health hazard for the causation of high disability and eventual morbidity cases with stable prevalence rates, even with treatment by the on-going multidrug therapy (MDT). Today, dapsone (DDS) resistance has led to fear of leprosy in more unfortunate people of certain developing countries. Herein, DDS was chemically conjugated with five phytochemicals independently as dapsone-phytochemical conjugates (DPCs) based on azo-coupling reaction. Possible biological activities were verified with computational chemistry and quantum mechanics by molecular dynamics simulation program before chemical synthesis and spectral characterizations viz., proton-HNMR, FTIR, UV and LC-MS. The *in vivo* antileprosy activity was monitored using the ‘mouse-foot-pad propagation method’, with WHO recommended concentration 0.01% mg/kg each DPC for 12 weeks, and the host-toxicity testing of the active DPC4 was seen in cultured-human-lymphocytes *in vitro*. One-log bacilli cells in DDS-resistant infected mice footpads decreased by the DPC4, and no bacilli were found in the DDS-sensitive mice hind pads. Additionally, the *in vitro* host toxicity study also confirmed that the DCP4 up to 5,000 mg/L level was safety for oral administration, since a minor number of dead cells were found in red color under a fluorescent microscope. Several advanced bioinformatics tools could help locate the potential chemical entity, thereby reducing the time and resources required for *in vitro* and *in vitro* tests. DPC4 could be used in place of DDS in MDT, evidenced from *in vivo* antileprosy activity and *in vitro* host toxicity study.

## Introduction

Multidrug therapy (MDT) with the sulphonamide drug, dapsone (4, 4′-diamino diphenyl sulfone or, DDS), along with antibiotics, rifampicin, clofazimine, and ofloxacin is the ongoing treatment option for leprosy, recommended by World Health Organization (WHO) today^1–4^. Its causative bacillus *Mycobacterium leprae* (*Ml*) had been under control by DDS in monotherapy since 1935. Today, DDS resistance has become one of the major obstacles for the control of leprosy with eventual treatment failure^2,5,6^. Consequently, the disease burden has reached to an alarming level of approximate 2,33,000 *Ml* cases globally; while, approximately 1,35,000 *Ml* cases with 5, 858 (~63%) cases of disability were recorded from India in 2016^3^. Nearly ~2,00,000 new cases are detected annually worldwide, with the highest prevalence in developing countries such as India, Nepal, Myanmar, Brazil, China, Madagascar, etc.^1,3^. DDS cannot be ordinarily replaced, despite its basic primary of morbidity from body intolerance, since it is the first-line drug for leprosy^7,8^.

**Figure 1:**
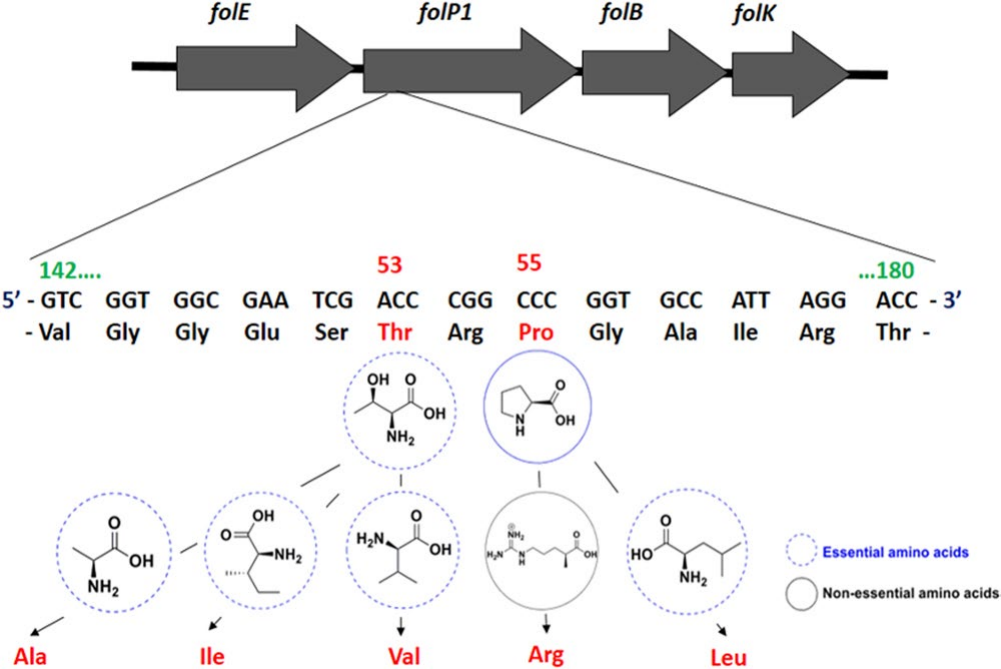
Diagrammatic presentation of the mutation event of *Ml*DHPS enzyme at *folP1* site confirming DDS resistance. At codons 53 and 55, the enzyme mutates sequences coding Ala or Ile or Val and Arg or Leu, respectively.

Dihydropteroate synthase (DHPS) is one of the nodal enzymes in the biosynthetic process of folic acid, which is essential for bacterial survival, while the human body derives folic acid from diets. DDS is an analog of the bacterial precursor para-aminobenzoic acid (pABA), which competitively inhibits the biosynthesis of bacterial folic acid, by targeting the putative DHPS enzyme^9,10^. Point mutations in the *folP1* gene-encoded DHPS, at codons 53 and 55 positions for Thr53 and Pro55, respectively (Fig. 1), are characteristic molecular signatures of DDS-resistance^2,11,12^. Recently, rifampicin was reported to be ineffective against *Ml*^13,14^. Thus, there is a look for an additional alternative potent agent(s) against leprosy in a relatively short time, as DDS-resistant bacilli spread fast. Continuing with wild-type (Wt) and mutant-types (Mt) forms of *Ml*DHPS as the identified targets^15^, the development of chemically altered DDS inhibiting bacterial folic acid synthesis had been organised^1^.

In the present study, based on structural suitability, DDS was chemically conjugated individually with five phytochemicals namely, 4-hydroxy coumarin, eugenol, salicylic acid, thymol and vanillin for aiming at the enhancement of its potency. The antileprosy efficacy and drug-likeness characteristics of proposed DDS-phytochemical conjugates (DPCs) were screened through chemoinformatics- and structural bioinformatics-tools viz., prediction of activity spectra for substances (PASS), molecular docking and molecular dynamic (MD) simulations with possible toxicity profile prediction in an ideal drug development approach, before the direct synthesis of conjugates. *Ml*DHPS enzyme represents the most suitable target for the possible development of the potential antileprosy drug. However, due to the lack of atomic resolution structure of *Ml*DHPS, the homology model of the protein was developed and validated by stereo-chemical quality tools before the molecular docking-simulation study with the conjugates. To cross-check the computational results and to get antileprosy physical activity, five proposed conjugates were synthesized and characterized by several spectral techniques, Fourier-transform infrared spectroscopy (FTIR), UV*-*spectroscopy, proton nuclear magnetic resonance (^1^HNMR), liquid chromatography-mass spectrometry (LCMS). Antileprosy activities of synthesized conjugates were tested in ‘mouse-foot-pad propagation’ (MFP) method using both DDS sensitive and resistant strains after inducing the disease by infecting mice; and the active most conjugate was selected for toxicity study using cultured human lymphocytes *in vitro*, isolated from umbilical cord blood (UCB).

## Materials and methods

### *Ml*DHPSs and DPCs 3D-structures modelling and validation

**Figure 2:**
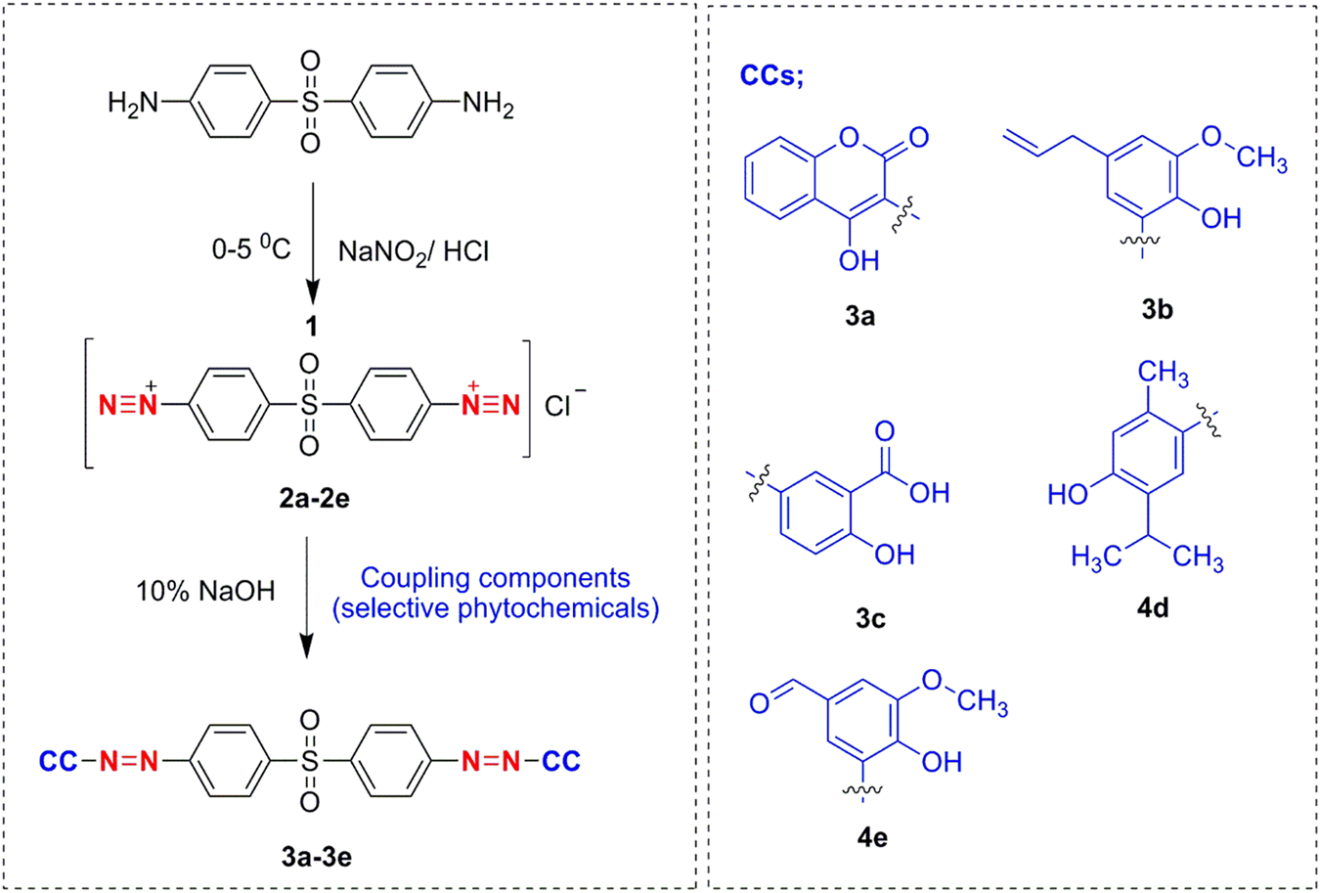
Stepwise synthesis process of DDS-phytochemical conjugates (DPCs).

Proposed five DPCs were designed based on azo-coupling reaction (Fig. 2) using ChemDraw Ultra 10.0 software similar to one reported earlier^1,9^. The retrieved DSS from the PubChem database (CID: 2955) and designed DPC structures were used in PDB file format, for molecular docking against the target *Ml*DHPS. The target protein sequence i.e., DHPS of *Ml* was obtained from the public domain UniProtKB (ID: P0C0X1). BLASTp (http://blast.ncbi.nlm.nih.gov/) and HHpred (http://toolkit.tuebingen.mpg.de/hhpred), suggested a consensus template. Later the target-template alignment generated using MultAlin (http://www.sacs.ucsf.edu/cgi-bin/multalin.py) (as shown in Fig. S1) was used for theoretical modelling of *Ml*DHPS with comparative modelling protocol of MODELLER9v18 tool^2^. The structural geometry of the generated *Ml*DHPS model was validated using the ‘Structure analysis and verification server (SAVE)’ and ProSA-web (https://prosa.services.came.sbg.ac.at/). After generation of this model, five mutations were introduced into the certified Wt structure at positions two positions Thr53 (T53I, T53A, and T53V) and Pro55 (P55R and P55L) generate five mutants as Mt targets, using BIOVIA Discovery Studio (BIOVIA DSV v4.5).

### Possible biological activity and toxicity prediction of the proposed DCPs

The predicted biological activities of the desired compounds were assessed on probable activity (Pa) and probable inactivity (Pi) values. Herein, antimycobacterium, antileprosy, antituberculosis, and antiinfective and activates of DPCs and DDS, through the PASS program (http://www.pharmaexpert.ru/passonline/). Based on the highest Pa value as suggested by PASS analysis, a compound can be selected and considered as the possible active drugable agent^9^. Additionally, ProTox (http://tox.charite.de/tox/) tool was employed to calculate the likely toxicity profiles, toxicity class and LD_50_ values of DPCs and DDS.

### **Molecular docking of DPCs against wild and mutant*Ml*DHPSs**

Molecular docking study was conducted using AutoDock 4.2, where both the Wt and Mt type model of *Ml*DHPS served as candidate target and DDS/DPCs treated as ligands. Kollman charges for target protein structures, whereas Geister partial charges were used for ligands through AutoDock Toolsv1.5. Binding site information based on the structural superposition with the close structural homolog co-crystallized with the drug was obtained from the published literature^16^. The grid was spaced around the binding site residues with a grid spacing 0.375 Å, with the threshold population size of 300 and the maximum number of energy calculations to 2.5 × 10^7^. The docked conformations from AutoDock were scored and clustered based on an RMSD cut-off of 2 Å. The top-ranked poses with higher biding free energy and ligand efficiency were subjected to all-atom molecular dynamics simulations in GROMACS to assess the structural integrity of the docked complexes.

### All-atom molecular dynamics simulation study

The dynamic behaviours of each *Ml*DHPS DDS/ DPCs complex was investigated using all-atom molecular dynamics simulations using Amber-99sb ILDN force field in TIP3 water models employed in GROMACSv5.1^1,17–19^. A three-step approach was employed for simulation studies, which include heating the system followed by equilibration and at the last the production run at 300 K for 40 ns for each system. The Linear Constraint Solver (LINCS) algorithm and the Particle Mesh Ewald (PME) method were employed to compute the electrostatic, hydrogen and covalent bond interactions for each protein-ligand complexes. The resultant trajectory was harvested at intervals of 20 ps for the analysis of pot dynamics.

### Trajectory analysis

Gromacs utility tools were employed for trajectory analysis. The quality assurance parameters like backbone RMSD, C-alpha root mean square fluctuations, a radius of gyration and intermolecular hydrogen bonds were computed using *gmx rms*, *gmx rmsf*, *gmx gyrate*, and *gmx hbond* utility toolkits of GROMACS^17,18^. Two-dimensional graphs depicting the dynamic stability were plotted using the Xmgrace tool. BIOVIA DSV was used to compute the inter-molecular interaction. In order to observe the highest amplitude records and correlated motions in complex systems, the most potent statistical technique, principal component analysis (PCA) was employed^1,17–19^. In this study, the covariance matrix of Cα-atoms of the complex systems was built using *gmx covar* tool. To comprehend the global motion of complexes (in phase space), the eigenvectors and eigenvalues were calculated, later projected into the phase space along the first two principal components (i.e., PC1 and PC2) using *gmx anaieg* tool.

### MM/PBSA binding free energy analysis

The calculations of binding free energy play a decisive role in understanding the dynamic interaction between ligands and the target protein. The molecular mechanics based MM/PBSA method used in *g_mmpbsa*v5.12, compatible with GROMACS, was used to compute the binding free energy (Δ*G*_*bind*_) of each system, using the snapshots from the MD trajectories. The binding free energy (Δ*G*_*bind*_) was calculated using the following equations:

1 $$\Delta {G}_{bind}={{\rm{G}}}_{complex}-{{\rm{G}}}_{protein}-{{\rm{G}}}_{ligand}={\Delta {\rm{E}}}_{MM}+{\Delta {\rm{G}}}_{sol}-{\rm{T}}\bigtriangleup {\rm{S}}$$

2 $${\Delta {\rm{E}}}_{MM}={\Delta {\rm{E}}}_{bonded}+{\Delta {\rm{E}}}_{nonbonded}={\Delta {\rm{E}}}_{bonded}+({\Delta {\rm{E}}}_{vdw}+{\Delta {\rm{E}}}_{ele})$$

3 $${\Delta {\rm{G}}}_{sol}={\Delta {\rm{G}}}_{polar}+{\Delta {\rm{G}}}_{nonpolar}$$

From the resultant 40 ns trajectories, 400 snapshots were extracted at equal intervals for binding energy estimations. The procedure for calculation of binding free energy was adopted from previous studies^1,17–19^.

### Synthesis and structural interpretation of DPCs

An ice-cool sodium nitrite solution of molarity equal to DDS was added dropwise with an 8 mL volume of concentrated HCl, and 8 mL water serially on an ice bath; after completion of the diazotization, the reaction was followed by pouring of ice-cool solution of 0.03 mol of individual phytochemicals in volumes of 20 mL 10% sodium hydroxide solution, as described earlier^1,9,17^. The resultant mixture was maintained at pH 5 to 6, and the obtained products were filtered and recrystallized with ethanol. Structures of the desired phytochemical conjugates were interpreted by several spectral techniques^1,9^. The open capillary method was used for the determination of melting points of the synthesized conjugates. The solvent-behaviour pattern of conjugates was studied by an UV-visible spectrophotometer. LCMS was performed for mass fingerprinting with pure conjugates analyzed through a Waters UPLC 1200 SQD-Mass Detector.

### **Dapsone resistance*Ml*and DHPS mutation confirmation**

DDS-sensitive and -resistant *Ml* strains were obtained from the Animal House Facility of Leprosy Centre, Karigiri, and all experiments with live animals were performed after approval of Institutional ethical committee, ‘Karigiri Research Committee of The Schieffelin Institute of Health Research & Leprosy Centre’, with relevant guidelines and regulations for use of mice (submission ID: 2014–2674, dated 18th Dec. 2014). These strains were passaged routinely in the hind foot pads of cross-bred (CBA) albino mice. Briefly, the sensitive strains of *Ml* were obtained from skin biopsies of leprosy cases, which were collected at diagnosis in the hospital and whose bacteriological index is >3+ at sites of skin lesions. These cases were later known to respond to MDT and were confirmed to have no mutations in *folP1* gene, corresponding to dihydropteroate synthase in *Ml* as the determinant of DDS resistance^20^. After grinding of biopsy samples in ‘mortar and pestle’ with the normal saline and a part of the suspension of bacilli, DNA extraction had been pursued using DNeasy Kits (Cat No: 69504, Qiagen Inc.). Extracted DNA samples were stored in 1X Tris EDTA buffer at −20 °C until further use. PCR was performed using *folP* detection primer with appropriate reactions and conditions for the confirmation of DDS resistance, as published^20^. DNA sequencing was carried out using the commercial facility (Scigenome Pvt. Ltd. Cochin, India) that confirmed the presence of mutations within the respective amplicons. The sequence reads were analyzed for quality and available mutations, using Molecular Evolutionary Genetics Analysis version 7.0 (MEGA).

### Assessment of antileprosy activity

Immuno-compromised (thymectomized irradiated, TR) CBA mice were used to multiply the identified strains of *Ml* to prepare sufficient inoculum for the experiments, as described^20,21^. The mice were maintained on a normal diet for 8–10 months for the infection to be established, and the counts were raised to a minimum of 1 × 10^6^ cells per hind foot pad, as per WHO guidelines. These mice were then inducted into the experimental groups for testing of the synthesized conjugates. For the current study, previously infection-established mice in the hind foot pads were chosen as detailed: five normal mice and five TR mice having DDS resistant strains, and five TR mice containing DDS sensitive strains. Those were divided into five groups for the to-be-tested drugs administered in the diet at the concentration of 0.01%, which was similar to the normal DDS concentrations used in screening for drug-resistant strains. The treatment duration lasted for 12 weeks, which was fixed based on the proportional bactericidal technique equating the WHO standard regimen of MB MDT in humans. After the treatment for graded durations, mice were euthanized and *Ml* cells were extracted from the hind footpads and enumerated subsequently. Enumerations were performed adopting methods that were employed for estimating the bacteriological index in human tissue samples^20,21^.

### Host-toxicity study of DPC4

The host toxicity of the effective most conjugate, DPC4 was carried out with cultured human lymphocytes purified from UCB^22^. DDS and DPC4 treated culture plates with different dosages (mg/L) against lymphocytes and incubated. The cytotoxicity was assessed using the acridine orange/ ethidium bromide (AO/EB) staining and the 3-[4,5-dimethylthiazol-2-yl] 2,5-diphenyltetrazolium bromide (MTT) assay and cells were checked by a fluorescent microscope (Magnus). Cell toxicity was assessed basing transformed probit values (Finney’s method) and those converted from percent lethality (PL) values, which were potted against corresponding log_10_ values of DDS and DPC4^22^.

## Results

### **Three-dimensional architecture of*Ml*DHPS**

The template i.e., the crystal structure DHPS of *M. tuberculosis* (PDB ID: 1EYE) had a sequence identity of 77% and query coverage of 100% was considered as the most appropriate template (Table S1), for comparative modelling of the target enzyme structure *Ml*DHPS (Figs. S1 and S2). Based on the Modeller scoring function and least Cα-root mean square deviation (RMSD) with the template, the best model was selected and optimized. The Ramachandran plot for the proposed model portrayed the Phi/Psi distribution of amino acids within the acceptable range, indicating its accuracy. A total of 94.4% residues fell in the most favored region, and the rest 5.6% residues were in the additional allowed area, while none of the residues fell in the disallowed region signifies the good quality of the constructed model (as shown in Fig. S3A). From ProSA-web, the Z-score energy profile of the generated model was −8.95, which matched with the Z-score range of experimentally derived template structures (as displayed in Fig. S3B; Table S2). The quality and consistency of the *Ml*DHPS model were validated using ProQ and METAMQAPII; the generated model was accepted for further studies based on the overall structural analyses (Table S2). Finally, the generated five mutants were used as targets against five newly designed DPCs as ligands in molecular docking investigation, to evaluate the strength of binding affinity using the software Auto Dock 4.2.

### Possible biological activity and toxicity prediction of the proposed DCPs

Concomitantly, antimycobacterial, antiinfective, antileprosy, antituberculosis activities of the proposed DPCs were recorded using the PASS program (as summarized in Table 1). From PASS prediction values, it was confirmed that the DPC4 had the most effective antileprosy ‘Pa’ values or Possible active values, 0.755 > 0.001, while the individual DDS ‘Pa’ values were 0.663 > 0.002. Similarly, all proposed DPCs had significantly effective ‘Pa’ values for antimycobacterial agents from which, DCP4 had the highest antimycobacterial ‘Pa’ value, 0.754 > 0.004; whereas, the DDS had ‘Pa value’ 0.647 > 0.007. Thus, PASS prediction indicated that all conjugates were active as antimycobacterial agents, and only the DPC4 was the most suitable conjugate against leprosy in the drug development endeavor. Furthermore, predicted toxicity, as toxicity class and lethal dose 50 (LD_50_) value too were assessed using the tool ProTox (Table 1). It was ascertained that DPC4 had LD_50_ values, 5000 mg/kg with toxicity class V (five); whereas the DDS had the LD_50_ value, 250 mg/kg with toxicity class III (three) (Table 1). Based on the determination of toxicity classes and computed LD_50_ values, it could be concluded that the described DPCs are comparatively less toxic and safe molecules; particularly, DPC4 could be a regarded as the active most antileprosy agent, with a low level of host toxicity and reasonably safe enough than the DDS.

**Table 1:** Predicted antimycobacterial, antileprosy, antituberculosic and antiinfective activities (Pa > Pi) and toxicity profiles of DPCs using prediction of activity spectra for substances (PASS) programme and ProTox tool.

DCs/DPCs	Prediction of activity spectra for substances (PASS)				Toxicity profile	
	Antimycobacterial	Antileprosy	Antituberculosic	Antiinfective	TC	LD_50_
DPC1	0.695 > 0.005	0.321 > 0.008	0.691 > 0.004	0.443 > 0.033	V	2100
DPC2	0.648 > 0.022	0.298 > 0.010	0.423 > 0.025	0.285 > 0.115	V	5000
DPC3	0.739 > 0.005	0.544 > 0.002	0.748 > 0.004	0.818 > 0.005	IV	2000
DPC4	0.754 > 0.004	0.755 > 0.001	0.728 > 0.004	0.740 > 0.005	V	5000
DPC5	0.693 > 0.005	0.371 > 0.005	0.690 > 0.004	0.292 > 0.109	V	5000
DDS*	0.647 > 0.007	0.663 > 0.002	0.610 > 0.005	0.751 > 0.005	III	250

DDS*, standard dapsone; DPC, dapsone-phytochemical conjugate; LD50, lethal dose value (mg/kg); TC, toxicity class.

### Analysis of docking-simulations

The resultant docked poses were screened to get the best conformation based on the highest docking score, ligand effectiveness and intermolecular hydrogen-bonds (as displayed in Table 2). Molecular docking analysis presented (represented in negative energy) that most of the DPCs had higher binding energy values than that of the DDS. From docking results, it was evident that the DPC4 (DDS with thymol) had a comparatively higher negative binding energy, −11.78, −10.84, −11.33, −11.38, −11.15 and −10.87 kcal/mol. In contrast, the individual DDS had −4.91, −4.91, −4.95, −5.01, −4.84, and −4.89 kcal/mol against the Wt and five Mt *Ml*DHPSs, respectively. Intermolecular analysis of DPCs with Wt and Mt *Ml*DHPSs displayed that the DPC4 complexes were stabilized through comparatively the highest numbers of intermolecular Hydrogen-bonds and electrostatic interactions. Therefore, DPC4 with Wt and Mt *Ml*DHPSs was considered to monitor the protein-ligand interaction and optimization, through molecular dynamics simulation in aqueous solutions.

**Table 2:** Docking scores and predicted toxicity class with LD_50_ values of DPCs along with dapsone against target enzyme *Ml*DHPS.

DCs/DPCs	No mutation	Mutation in 53 position			Mutation in 55 position	
	Receptor-1(wild type)	Receptor-2(Thr to Ile)	Receptor-3(Thr to Ala)	Receptor-4(Thr to Val)	Receptor-5(Pro to Arg)	Receptor-6(Pro to Leu)
DPC1	−10.816	−10.614	−10.630	−10.676	−10.512	−10.305
DPC2	−10.762	−10.480	−10.497	−10.547	−10.793	−10.403
DPC3	−10.754	−10.249	−10.047	−10.523	−10.438	−10.249
DPC4	−11.785	−10.844	−11.339	−11.381	−11.155	−10.875
DPC5	−10.531	−9.797	−10.164	−9.525	−9.552	−10.055
DDS*	−4.910	−4.910	−4.950	−5.010	−4.840	−4.890

### Analysis of MD trajectories and intermolecular H-bond analysis

**Figure 3:**
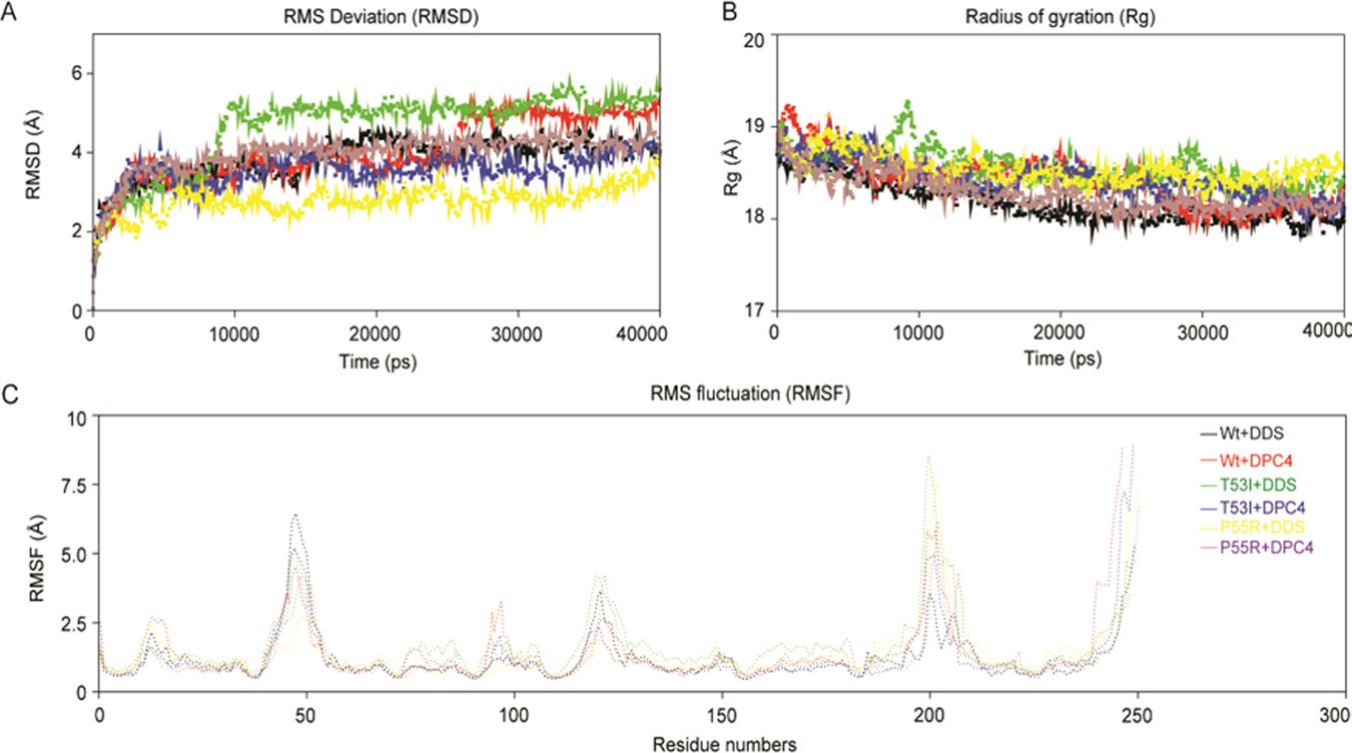
Conformational stability of *Ml*DHPS‐DDS/DPC4 complexes during 40‐ns time period indicated in different color graph. **(A)** RMSD of *Ml*DHPS‐DDS/DPC4 complexes; **(B)** Rg of *Ml*DHPS‐DDS/DPC4 complexes; **(C)** Cα‐RMSF profile of the target, *Ml*DHPS‐DDS/DPC4 complexes during 40 nano second molecular dynamic simulation.

MD simulations offer to probe the behavioral dynamics of bio-macromolecules including proteins and systems at length scales ranging from nanometres to close to a micrometer and concomitantly on microsecond timescales. In this study, the employed all-atom MD simulations of protein-ligand complexes were considered to explore in detail of interactions of DPCs/ DDS with both Wt and Mt *Ml*DHPS enzyme individually at a molecular scale. To measure the dynamic stabilities of each complex, the backbone RMSD, Rg, Cα-RMSF and inter-molecular H-bonds from the resultant trajectories were computed. The trend in RMSD of all systems rapidly increased up to 5 Å during the initial ten nanoseconds (ns) and after that, a relatively constant value ~2.47–3.37 Å was maintained with little exceptions for the Wt *Ml*DHPS with DPC4 and DDS bound T53I (Fig. 3A). During the first 1–35 ns *Ml*DHPS with DPC4 and the T53I with DDS systems displayed slightly higher RMSD as compared to the other systems; however, both systems attained stable RMSD after 30 ns. Unlike the RMSD, the other stability parameter Rg followed slightly a different trend, where all complexes had decreased the radius of gyration (Rg of ~18.57 Å), indicating the compactness of the systems till 40 ns (Fig. 3B). The flexibility among the residues upon ligand binding can be inferred from the trend in average root mean square fluctuation (RMSF) of Cα-atom with respect to time. It can be witnessed that all the protein-ligand complexes followed a more or less similar trend in RMSF with minute exceptions in a few cases (Fig. 3C). The 3_10_ helix, loops adjoining helix and strand along with variable loops, with some higher peak values displayed a comparatively high degree of fluctuations in the respective Cα atoms in all systems signifies the flexibility of these residues. A reason behind the recorded higher RMSF values with certain residues and Cα-RMSF of binding site residues were the higher fluctuations; those indicated corresponding close participation in ligand/ drug binding events.

**Figure 4:**
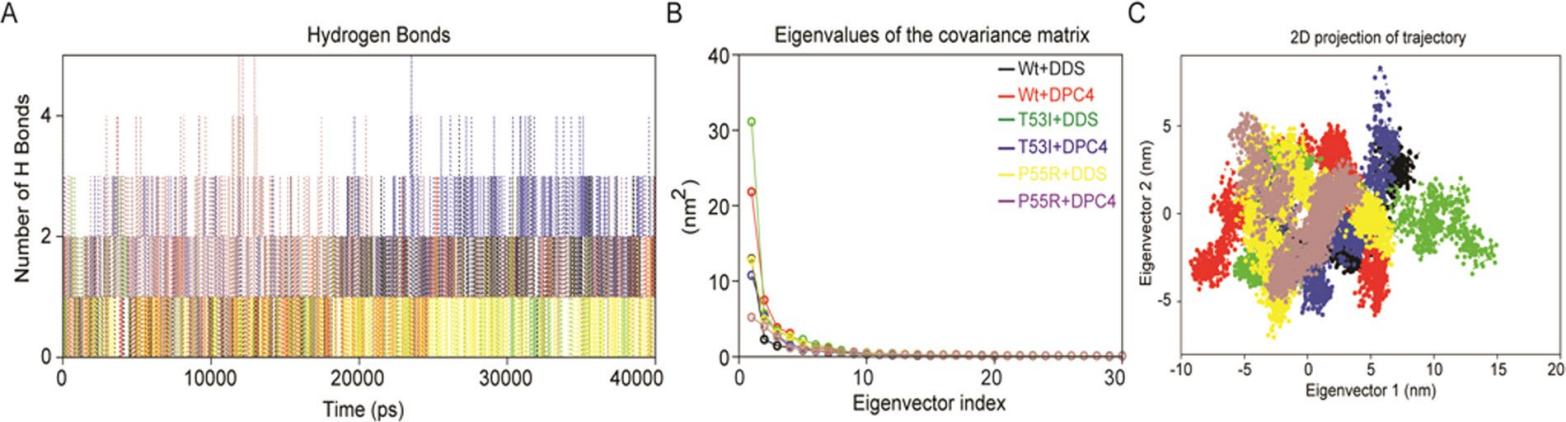
(**A**) Stability of *Ml*DHPS‐DDS/DPC4 complexes inferred through intermolecular Hydrogen‐bonds; (**B)** Principal component analysis of the DCP4/DDS interacted with the target enzyme, *Ml*DHPS of both wild‐type and mutant‐type structures. Eigenvalues for the *Ml*DHPS‐DCP4/DDS bound models as a function of eigenvector and the plot displays the eigenvalues of only the first 20 eigenvectors; and (**C)** The cloud represents the 40‐ns trajectories projected onto the first two eigenvectors, EV1 and EV2. The X‐axis and Y‐axis show the projection of the structures of the backbone atoms in the molecular dynamics trajectories of complexes onto the phase space defined.

For scrutinization of intermolecular H-bonding patterns in *Ml*DHPS-DDS/DPC4 complexes, an intermolecular H-bond analysis was performed for six complex systems. It was evident that six systems displayed differential patterns of H-bonding (Fig. 4A). H-bond analysis between the DPC4 with Mt (P55R) DHPSs yielded discernibly higher numbers of H-bonds (~4.12), followed by those of T53I with DPC4. Despite there was a significant deviation in H-bonds during the initial 20 ns, stability was maintained untill 40 ns (Fig. 4A). In some cases, for example, DPC4 with Wt and DDS with Mt, a few H-bonds were seen to be broken during MD, which was compensated through newer electrostatic and hydrophobic contacts. Adaptations in intermolecular H-bonds and other non-bonded interactions were witnessed before and after MD simulations in all protein-ligand systems impeccably, which corroborated the similar analysis of RMSF values; the existence of differences in residue flexibility of complexes was demonstrated.

### Principal component analysis

**Figure 5:**
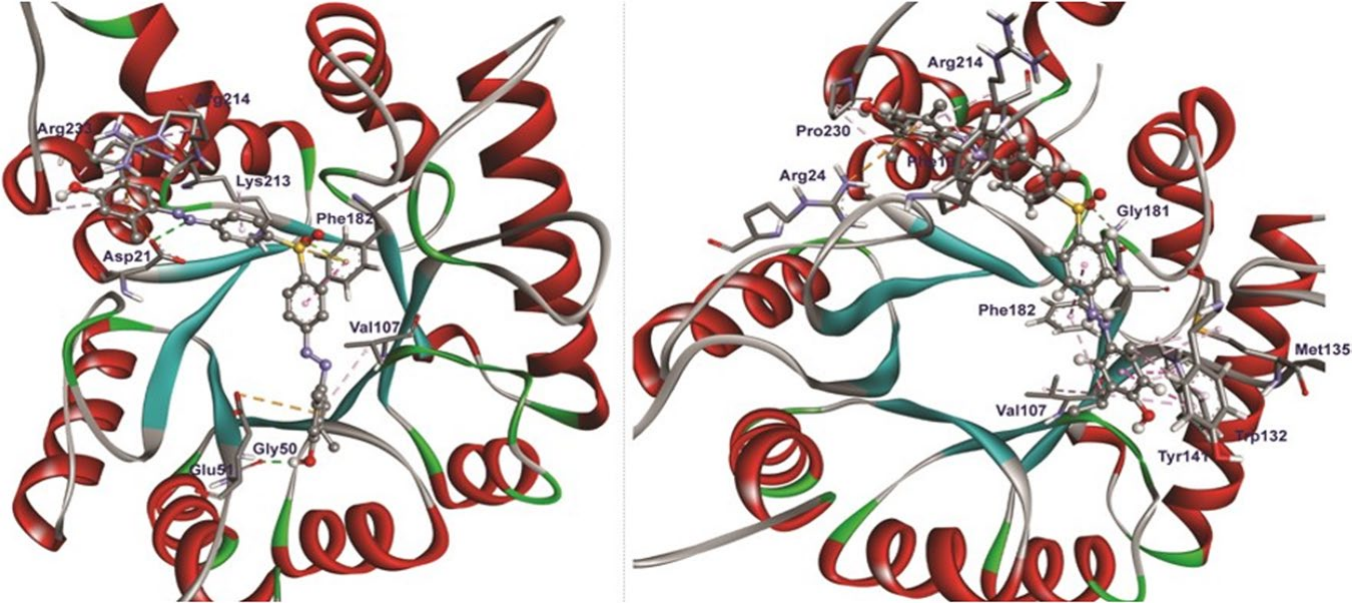
Molecular interactions of DPC4 with mutant *Ml*DHPS (P**55**R) during docking. The interactions image was generated using BIOVIA DSV and interacted amino acids are highlighted.

The snapshots extracted from complex trajectories were projected onto the phase space to obtain a range of eigenvectors (EVs) or principal components (PCs), which represent the vectorial illustration of each single element of the motion indicating the direction of individual movement. Several studies have shown that the top of the first few eigenvectors (EVs) capture bulks of the internal motions; however, the first two eigenvectors were considered (EV1 and EV2), which accounted for values more than 80% of overall recorded motions projected into the phase space. As it is, eigenvalues obtained from each EV describe the active movement of each component to the motion. The obtained steep curves of eigenvalues in ‘plots of eigenvalues against the respective eigenvectors’ (Fig. 4B), covering around 92.1, 89.3, 87.5, 82.0, 94.8 and 84.2%, progressively, the backbone motions were recorded increasing initially by 30 eigenvectors. Movements of both states of Wt and Mt *Ml*DHPS complex systems in phase space were obtained by projecting the trajectories that were acquired onto the first two EVs (Fig. 4C). Least differential scattering images of atoms were observed in all cases, which specify the occurrence of least conformational changes in the complex structures, in agreement with MD analysis. The overall flexibility of DPCs/ DDS bound structures was inferred by the trace of the diagonalized covariance matrix of central chain atoms. The T53I with DDS (trace value, 28.47 nm^2^) and Wt with DPC4 (trace value, 28.37 nm^2^) complex systems displayed a little higher scattering of atoms with high trace values in each, with eventual confirmation of the overall increased flexibility in case of Wt-complex than Mt form having been well supported by RMSF analysis. Furthermore, to explore the conformation heterogeneity in the ensemble of complex structures obtained from each trajectory, the GROMOS clustering algorithm was employed with an RMSD cut-off at a value of 0.2 nm. Indeed, the RMSD of clusters were within the range of ~0.123 to 0.178 nm, and the clusters with the maximum numbers of representatives were visually inspected for protein-ligand interactions using BIOVIA DSV (Fig. 5). It was concluded as the DPC4 strongly interacted with the Mt *Ml*DHPS as compared to DDS.

### Pre- and Post-MD docking simulation and MM/ PBSA binding free energy analysis

Obtained from the clustering approach, the representative structures were used for the interactive analysis of DPC4 with Wt and Mt *Ml*DHPSs, individually (Table S3). Before MD, a total of two H-bonds were found between Wt *Ml*DHPS with DPC4 at the amino acid position, Asp21 with 3.15 Å and Gly50 with 2.0 Å distance, respectively. On another hand after MD, three H-bonds were formed with Arg139, Tyr141, and Arg139 having atomic distances, 2.34, 1.67 and 3.17 Å, respectively (Table S3B). Furthermore, pi-pi stacking, pi-sulfur, and pi-alkyl interactions were also noticed with Arg214, Glu51, Asp21, Phe182 and Pro230, before MD in the Wt *Ml*DHPS-DPC4 complex. Furthermore in the Wt complex, more pi-cation interactions with Phe19, Tyr141, Phe19 and Tyr141, pi-anion with Glu51, more pi-alkyl interactions with Phe19, Trp132, Tyr141, Pro140, and Ala112 were observed. In the Mt *Ml*DHPS (P55R)-DPC4 complex, a total of two H-bonds were observed in Asp21 with the interaction distance, 2.90 Å and Gly50 with the distance 2.13 Å. Along with pi-cation in Arg214, pi-anion in Asp21 and Glu51, pi-sulfur in Phe182, pi-pi stacked in Phe182, pi-alkyl in Pro230 and Lys213 positions were observed before MD (Table S3F). While Gly181 formed H-bond interactions with DCP4, Arg24 had interaction as pi-cation, and Trp132 as pi-pi stacked, Phe182 as pi-pi along with Phe19, Trp132, Trp132, Tyr141, Phe182, Arg214, and Met135 having been contributed as pi-alkyl interactions with an average of ~3 to 4 Å distances, seen after MD simulations. Thus, Phe19, Val107, Tr132 and Tyr141 could be stated as important amino acids towards the strong molecular inhibition of DPC4 with the modelled *Ml*DHPSs pterin binding pocket. The cross verification with the selected templates, the pterin binding pocket with amino acids had the significant role of interaction with drugs, corroborating the earlier report^16^. Additionally, it can be summarized that apart from intermolecular H-bonds, the other non-bonded contacts involving hydrophobic and electrostatic contacts play active roles in the *Ml*DHPS-DPC4 mediated interaction (Fig. 5).

To understand the driving force behind molecular recognition of DPCs/DDS against *Ml*DPHSs, the binding free energies were estimated using snapshots from MD trajectories using the MM/PBSA method. The discrete energy components, E_*MM*_, G_*polar*_, and G_*nonpolar*_ of each complex contributing towards binding free energy were summarized (Table S3). Computed binding free energy values of DDS to Wt and Mt *Ml*DHPS complexes were within the range of −26.92 ± 0.46 to −50.10 ± 0.85 kJmol^−1^; while for the designed DPCs had energy values in the range of −112.30 ± 1.02 to −166.29 ± 1.93 kJmol^−1^, respectively. The predictable negative electrostatic interactions were for Wt/Mt *Ml*DHPSs-DDS/DPCs interaction symbolizing the electrostatic attractions between receptors and corresponding ligands (Fig. S4). The polar contributions for solvation were allocated positive values ranges within 84.76–299.53 kJ mol^−1^. DPCs possess comparatively higher negative free energy values for the Wt as well as, Mt *Ml*DHPSs, as compared to DDS, concluded from the binding free energy analyses.

### Synthesis and characterization of DPCs

After the detailed computational analyses and good responses from there, all five proposed DPCs were synthesized by the azo-dye coupling method, and structural conformations of individual conjugates were further ascertained. From the FTIR spectra of conjugates, DPC4 was seen with sharp absorption bands at 3366, 1355, 1145 and 1499 cm^−1^, due to the presence of phenolic hydroxyl -OH(str.) of thymol, asymmetrical and symmetrical stretching of the sulfone radical SO_2_(str.) and the newly inserted –N = N-(str.) entity, respectively; consequently confirming the described structural modifications of DDS (Fig. S5). Furthermore, solvatochromic effects of DPC4 in ethanol had been recorded having a strong bathochromic shift (λ_max_384), due to extensive chromophoric conjugation of thymol as compared with the precursor DDS. From the proton NMR of the synthesized DPC4 on DMSO-*d*_6_, it could be inferred that 8 aromatic double doublets protons are present at δ 8.17–8.39 ppm with respect to the DDS nucleus, and five singlet protons including two aromatics, two methyl and one board singlet OH proton of thymol were present that appeared at δ 7.09, 7.64, 2.34, 1.25 and 9.69 ppm, respectively (Fig. S6). From LCMS study, the single quadrupole mass detector analysis confirmed predicted molecular formulae of conjugates; notably, the molecular fingerprint of DPC4 at m/z 566.13 in 1.20 min (retention factor) with a percent of area 95.81%, actively assigned the predicted chemical formula, C_32_H_34_N_4_O_4_S (Fig. S7). Similarly, the rest four DPCs were characterized (Supporting Information).

### **Dapsone resistance*Ml*and DHPS mutation confirmation**

With preliminary demographic and clinical characteristics of the leprosy cases, DNA was extracted from skin samples, using the lysis protocol for verification of the DDS resistant strain of *Ml*. The ‘WHO Guidelines for Sentinel Surveillance Study of Drug Resistance in Leprosy’ was followed for detecting mutations using the mouse-foot-pad experiment with ancillary PCR technique, using the specific primer of *folP* gene for *Ml*. After confirmation of the DDS resistance band through PCR (Fig. S8), the presence of mutations in 53 and 55 coding positions within the amplified product was confirmed after DNA sequencing (Fig. S9). Sequence data were analyzed using MEGA Version-7 (Molecular Evolutionary Genetics Analysis) and Sequencer V. 5.4.6.

### Antileprosy activity evaluation of DPCs

DDS -sensitive and -resistant infected mice were administered with DPCs in the diet at the concentration 0.01%, which is similar to the average DDS concentrations used in screening for drug-resistant strains. The treatment duration was fixed for 12 weeks, based on the proportional bactericidal technique equating the WHO standard regimen of MB-MDT for humans. After the treatment duration, mice were euthanized and *Ml* bacilli cells were extracted from the hind footpads and enumerated. From *in vivo* evaluation, the DPC4 was found as the effective-most conjugate with a one-log reduction in resistant infected mice foot pads, and no bacilli were found in the sensitive hind pad of infected mice within 12 weeks of treatment. Blithely, the rest four conjugates reduced one-log in bacilli counts only in the 12 weeks of treatment similar to that of the DPC4 (Table 3). However, further pharmacological validation is required for the replacement of DPC4 in place of DDS in the presently used MDT regimen.

**Table 3:** Antileprosy activity of 5 DPCs along with individual dapsone (in concentration 0.01% mg/kg) in mouse foot-pad propagation method.

Dapsone/DPCs(0.01%)	Normal/TR mice	Resistant/Sensitive strain	Bacilli cell counts before treatment	Bacilli cell counts after treatment
DPC1	Tr	R	2.3*10^6^	1.7* 10^6^
	N	R	8.2*10^5^	5.7* 10^5^
	Tr	S	4*10^6^	4.7* 10^5^
DPC2	Tr	R	1.3*10^6^	5.6* 10^7^
	N	R	1.6*10^6^	5.6* 10^5^
	Tr	S	6.7*10^6^	1.4* 10^5^
DPC3	Tr	R	1.3*10^6^	2.3* 10^6^
	N	R	1.6*10^6^	2.6* 10^6^
	Tr	S	6.7*10^6^	2.2* 10^5^
DPC4	Tr	R	5.8*10^5^	2.6* 10^4^
	N	R	6.1*10^5^	4.8* 10^5^
	Tr	S	5*10^5^	no growth
DPC5	Tr	R	5.8*10^5^	2.5* 10^6^
	N	R	6.1*10^5^	6* 10^5^
	Tr	S	5*10^5^	6.4* 10^6^
DDS	Tr	R	1*10^5^	1.3* 10^5^
	N	R	7.4*10^5^	1.4* 10^6^
	Tr	S	4*10^6^	1.3* 10^6^

The recorded bacilli cell counts after 3 months of treatment.

N, normal; R, resistance; S: sensitive; Tr: immunocompromised (thymectomized irradiated).

### Host toxicity of the most active DPC

**Figure 6:**
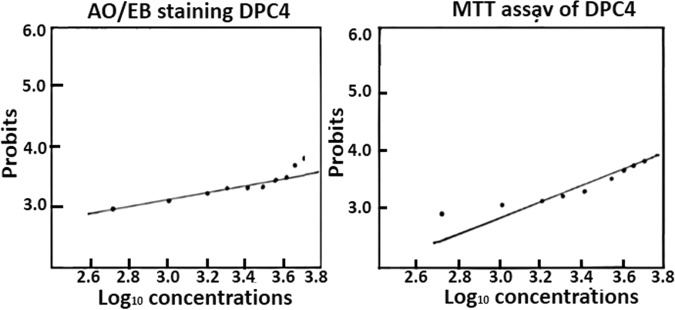
The log_10_ concentration values were determined taking probit points of cytotoxicity ascertained by AO/EB staining and MTT assay; and probits were plotted for DPC4 during toxicity study with lymphocytes, for establishing toxicity.

Indeed, toxicity study is one of the critical parameters for authenticating a newly synthesized chemical entity as a prospective drug candidate. The host toxicity of the active conjugated agent, DPC4 was studied using human lymphocytes from UCB, as it is a waste of blood from the hospital after infant birth. In the *in vitro* toxicity study with the concentration up to 5000 mg/L of DCP4, a minor number of dead cells were found in a fluorescent microscope. Cell toxicity was analyzed basing on transformed probit values (Finney’s method), and those converted from percent lethality (PL) values (Table S4), potted against corresponding log_10_ values of DDS and DPC4 for a probit plot (Fig. 6). Indeed, the computational toxicity assessment was corroborated by the toxicity study in cultured human lymphocytes^22^. Thus, this systematic novel finding could be taken as an ideal approach towards the newer development of a conjugated against drug-resistant *Ml*.

## Discussion

DDS resistance in leprosy was known during its implementation in the monotherapy treatment in 1966. This problem was addressed to a certain extent after the introduction of MDT by WHO in the year 1983, when the combinatorial regimens were used against paucibacillary and MB leprosy^1,2^. Nevertheless, with the emergence of resistance of DDS and rifampicin in recent years, the rates of relapsed leprosy cases with resistance to MDT gradually increased, posing a threat to public health. In this context, the introduction of a new drug or modification of existing drugs in MDT is only a possible option to control drug-resistant leprosy, since no new drug candidate against leprosy is available, approved by WHO and FDA in the last two decades. Due to the associated complications and time killing studies of drug development against leprosy, failures are plenty for revising the current MDT in replacing DDS^23–25^. Thus the same three drugs, DDS, rifampicin and clofazimine were continued in use with an altered dose schedule since 1992. However, only the treatment interval for MB leprosy was reduced from 24 to 12 months through repurposing drug combination attempts with the new additions of ofloxacin and minocycline in rifampicin resistance case in between 1982–1998^24,25^. However, the revised MDT too has been a persisting failure for DDS and rifampicin-resistant cases^1,2^; nevertheless, the time-consuming MFP method has been the conventional gold-standard method in detection of drug resistance pattern and antileprosy activity of a new drug, since the bacilli cannot be grown *in vitro*. When viewed from trenches of the management of this deadly disease, locating some clinically active DPC before synthesis, the use of a bandwagon of advanced bioinformatics tools is a dire necessity to save time and resources. In the present study, DPCs were developed as probable effective antileprosy agents with the help of the azo-coupling reaction method. The recorded computational results of PASS and toxicity profile predictions, RMSD, Rg and inter-molecular H-bonds analyses, MM/PBSA binding free energy values, along with van der Waals force accounts, electrostatic and SASA energy estimations approaches herein helped to get a decisive role in promoting the DPC4 as the suitable for synthesis as future agent in MDT. Indeed, the possible biological active values as an effective antileprosy agent from PASS prediction and the binding free energy score against the Mt *Ml*DHPSs proclaimed that the DPC4 was significantly higher than those of DDS.

However, the generated simple scoring functions or docking scores obtained through docking program often disregards the noteworthy energetic contributions as solvation free energy, which is not ideal for the calculation of the binding affinity and correct binding pose of any small molecule or ligands. Hence, the exact ligand-binding pose was considered by integrating the docking program, all-atoms MD simulations and the binding free energy MM/PBSA method, since the latter approach had been seen as a practical and trustworthy approach to model molecular recognition mechanisms involving receptor-ligand binding interactions^17–19^. Amongst the popular classical simulation methods, the MM/PBSA method had been considered a valid and reliable way to model molecular recognition, including receptor-ligand binding interactions^1,20,21^. Structural characteristics of *Ml*DHPS-DPC4 and *Ml*DHPS-DDS complexes, obtained from attempts with AutoDock, performed explicit solvent MD simulations using the GROMACS package as illuminated. To quantify the structural dynamics and intrinsic stability of the receptor-ligand complexes, backbone RMSDs, Cα-RMSF, Rg, and intermolecular H-bonds were computed. The dynamics analysis of these complexes suggested the complexes generated through docking retain the most of properties during MD. From free energy decompositions analysis it could be ascertained that the predominant force of the binding process for the DDS/ DPCs with Wt/ Mt *Ml*DHPSs correlated van der Waals energy. Because the negative van der Waals energy occurs in all simulation systems, it can be considered that van der Waals plays a substantial role in forming stable complexes. In addition, the electrostatic energy and SASA energy accounts for binding free energy. This signifies the designed DPCs might be more superior to that of DDS, and the stated conclusion needs further *in vivo* investigation for its validity. However, the computed binding free energies cannot be taken absolute due to lack of *in vivo* experiments; nonetheless, the binding free energy study (without entropic contribution) has provided evidence on the molecular recognition of receptor *Ml*DHPSs with DDS and DPCs. Now a day, MD simulation methods have become a monotonous tool in modern drug discovery processes. These techniques, for explicit structural flexibility and entropic effects, help to estimate thermodynamic features and associated underlying kinetics with receptor-drug recognition and binding precisely with novel computational algorithms. Hence more future studies, including novel MD methods may help in the discovery of novel drug candidate(s) for the protocol of multidrug therapy against leprosy in the future^26–28^.

In mainstream drug development, the conjugation of a secondary plant metabolite having suitability for chemical compatibility with an inactive drug was considered as an ideal approach^9^. A similar type of work had been examined against cancer treatment as ‘antibody-drug’ or ‘antibody-cyanobacterial chemical’ pairs have gained importance as antimalarial and antibacterial drug development earlier^29–33^. Thus, this chemical conjugation is a new trend in antimicrobial drug development to enhance the activity and pharmacokinetic properties^34–38^.

## Conclusion

The obtained MFP results should be a milestone for the DPC4 in reducing one-log DDS resistant bacilli-population *in vivo* in three months of treatment only, and no bacilli were found in treating DDS sensitive *Ml*, simultaneously. The cytotoxicity study also helped to state that, after the conjugation of a phytochemical to DDS, the DDS-conjugate enhanced antileprosy activity *in vivo* with reduced host toxicity pattern *in vitro*. Overall, the systematic computational analysis, coupled with medicinal chemistry, the conjugation of a phytochemical with a clinically inactive mainstream drug, could be a new trend in the current drug discovery, as here.

## Supplementary information

Supplementary information.
