# Supplementary material for: Computer-aided synthesis of dapsone-phytochemical conjugates against dapsone-resistant Mycobacterium leprae
Source: Sci Rep. 2020 Apr 22;10:6839. doi: 10.1038/s41598-020-63913-9 (PMC7176699; doi:10.1038/s41598-020-63913-9)

**Supporting Information**

**Computer-aided synthesis of dapsone-phytochemical conjugates against dapsone-resistant *Mycobacterium leprae***

Shasank S. Swain^a, b^, Sudhir K. Paidesetty^c^, Budheswar Dehury^b, d^, Madhusmita Das^e^, Sundeep C. Vedithi^e, f^, Rabindra N. Padhy^a,*^

^-------------------------------------------------------------------------------------------------------------------------------------------------------------------^

**Supporting Figures**


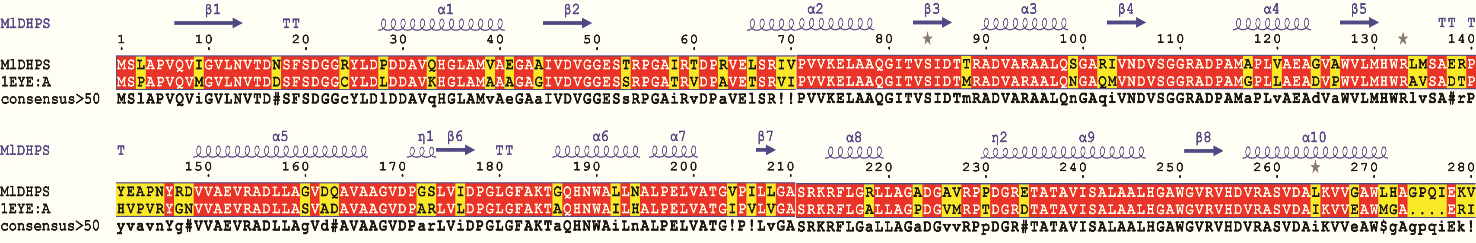


**Figure S1.** The pair-wise alignment of the *Ml*DHPS and the selected template 1EYE (crystal structure DHPS from *Mycobacterium tuberculosis*) was constructed using Multalign and ESPript. The secondary structural elements were identified using ESPript. The α-helices, η-helices, β-sheets and strict β-turns are denoted α, η, β and TT respectively. Similar amino acids are highlighted in boxes, and completely conserved residues are indicated by white lettering on a red background.


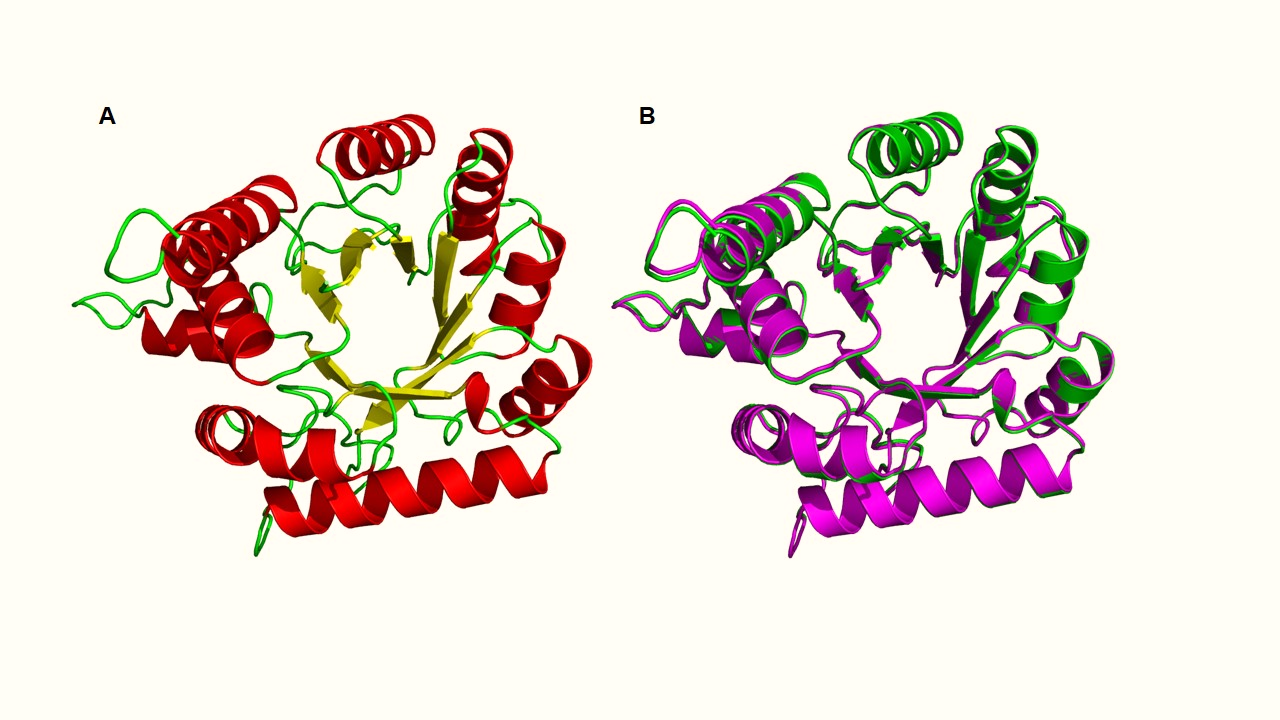


**Figure S2.** (**A)**, The solid ribbon form of newly generated three‐dimensional homology model *Ml*DHPS, colored based on its secondary structure elements. (**B)**, Structural superimposition of modeled *Ml*DHPS and the crystal structure of template (PDB ID: 1EYE), generated using PyMOL.


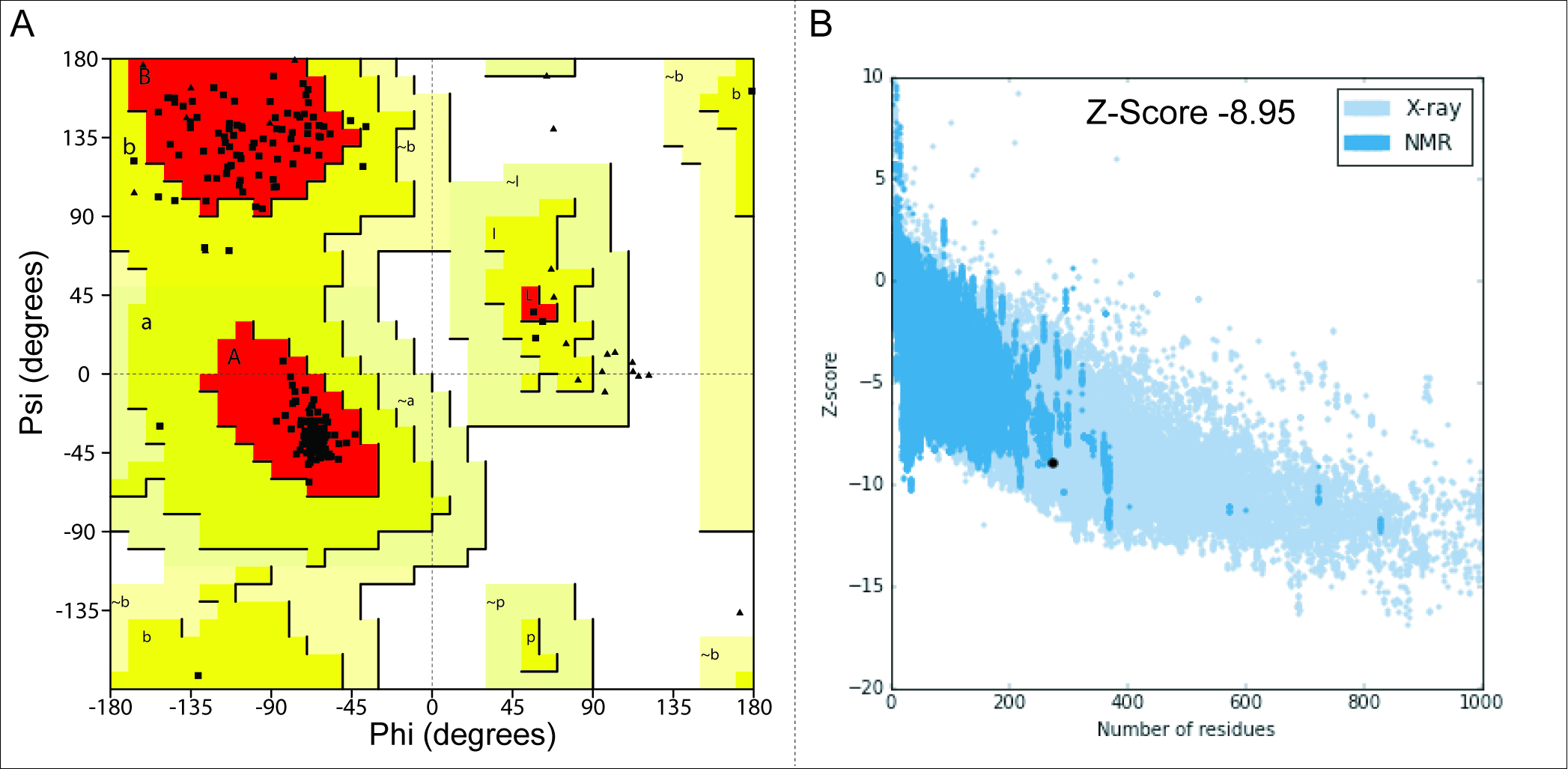


**Figure S3.** Ramachandran plot and overall quality of the proposed *Ml*DHPS model was assessed using Procheck Program and ProSA-Web tool.


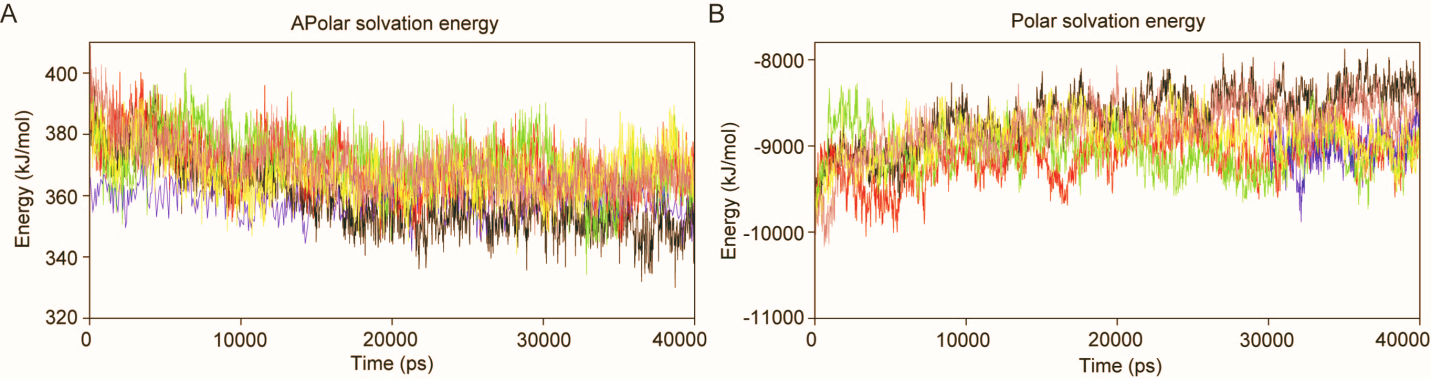


**Figure S4.** Energetic contribution (polar and apolar) of DPC4 with Wt and Mt *Ml*DHPS obtained through molecular mechanics/ Poisson‐Boltzmann surface area method.


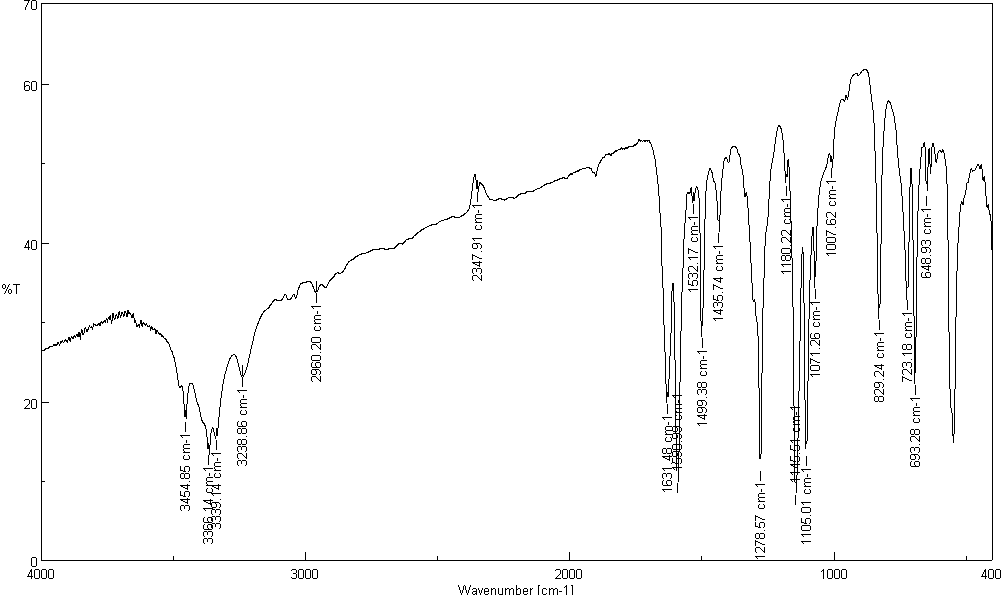


**Figure S5**. FTIR spectra of DPC4.


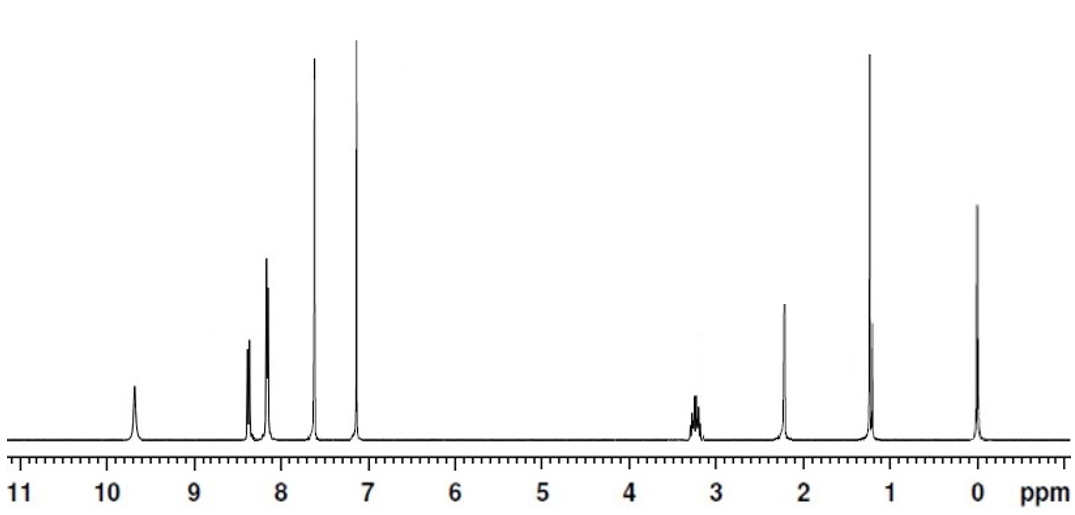


**Figure S6.**  Proton NMR spectra of DPC4.

**
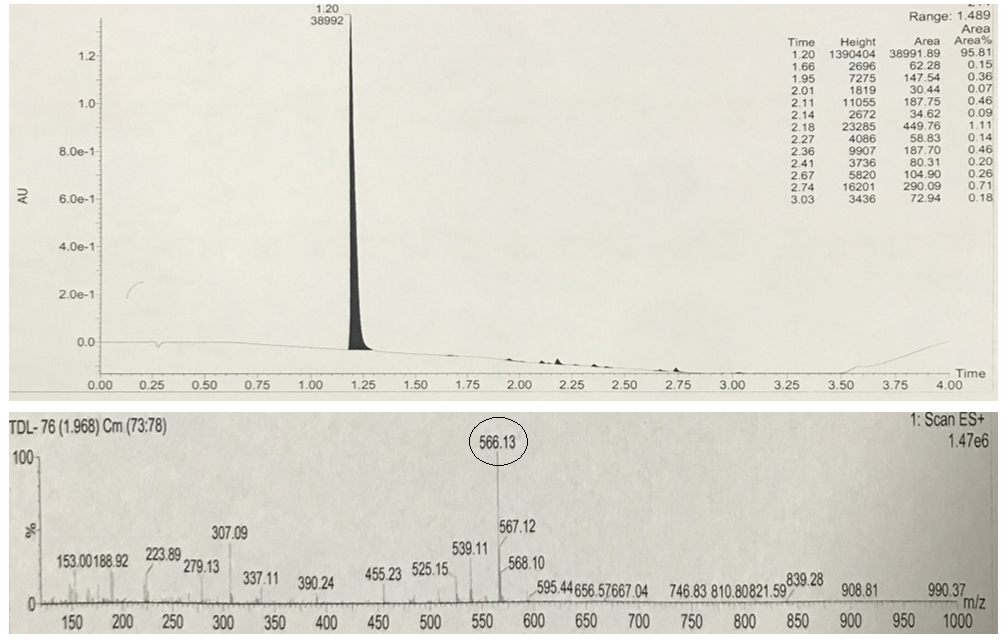
**

**Figure S7.** LCMS spectra of DPC4.


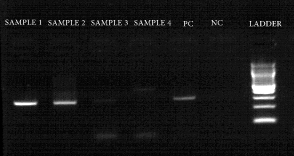


**Figure S8.** DDS resistance conformation by PCR.


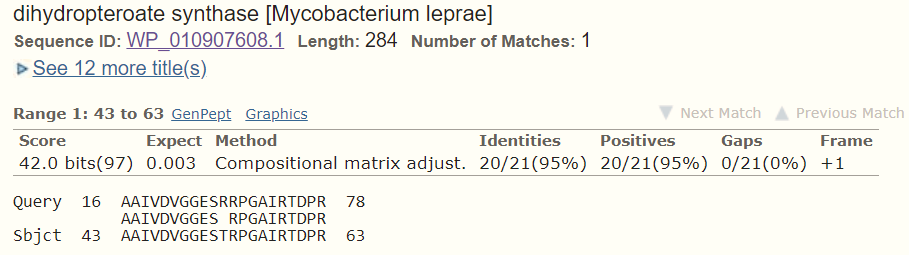


**Figure S9.** Mutation analysis after sequencing position (picture page of BlastP tool).

**Supporting Tables**

**Table S1:** Identification suitable template structure (PDB ID: 1EYE) for homology modelling of target enzyme, *Ml*DHPS through several bioinformatics tools.

| **Tool** | **Parameters used for identification of suitable template structure** | | | |
| --- | --- | --- | --- | --- |
|  | Query coverage | % Identity | E-value | Rank |
| BLASTp | 100 | 77 | 6e-143 | 1^st^ |
| CS-BLAST | 86 | 77 | 8e-97 | 1^st^ |
| CPHmodels | 94.7 | 75.5 | 1e-102 | 1^st^ |
| HHpred | 100 | 78.21 | 1.3e-50 | 2^nd^ |
| Phyre2 | 100 | 79 | - | 1^st^ |
| Raptor X | 84 | 71 | 4.47e-08 | 1^st^ |
| SWISS-MODEL | 100 | 79 | - | 1^st^ |

-, not found

**Table S2:** Comparative analysis of various model validation scores of the modelled *Ml*DHPS and its close structural homolog DHPS of *Mycobacterium tuberculosis* (PDB ID: 1EYE).

| **Tools** | **Model validation parameters** | **Scores** | |
| --- | --- | --- | --- |
|  |  | **Target (*Ml*DHPS)** | **Template (PDB ID:1EYE)** |
| PROCHECK | Most favored region (%) | 93.9 | 94.0 |
|  | Additional allowed region (%) | 6.1 | 6.0 |
|  | Generously allowed region (%) | 0.0 | 0.0 |
|  | Disallowed region (%) | 0.0 | 0.0 |
| Verify3D | Averaged 3D–1D score >0.2 | 88.69 | 92.97 |
| ERRAT | Overall quality | 85.71 | 99.19 |
| ProSA | Z-Score | -8.88 | -8.60 |
| ProQ | LG score | 4.043 | 6.350 |
|  | MaxSub | 0.324 | 0.615 |
| MOL Probity | Cβ deviations >0.25Å | 0.82 | 3.90 |
|  | Residues with bad bonds (%) | 0.98 | 0.00 |
|  | Residues with bad angles (%) | 0.47 | 0.27 |

**Table S3:** The binding free energy of DDS/ DPC4 with wild and mutant type *Ml*DHPS computed using MM/PBSA method.

| **BE parameters**  **(kJ/mol)** | **Wild Type (Wt)** | | **Mutant Type (Mt)** | | | |
| --- | --- | --- | --- | --- | --- | --- |
|  | **Wt-DDS** | **Wt-DPC4** | **Mt(T53I)-DDS** | **Mt(T53I)-DPC4** | **Mt(P55R)-DDS** | **Mt(P55R)-DPC4** |
| van der Waal energy | -130.39 ± 0.34 | -228.13 ± 0.40 | -98.95 ± 0.29 | -262.20 ± 0.74 | -121.22 ± 0.31 | -239.42 ± 0.57 |
| Electrostatic energy | -133.233 ± 0.51 | -150.11 ± 0.71 | -24.44 ± 0.40 | -80.64 ± 0.53 | -130.50 ± 0.36 | -40.89 ± 0.37 |
| Polar solvation energy | 252.93 ± 0.83 | 299.53 ± 1.08 | 84.76 ± 1.06 | 241.49 ± 0.91 | 238.58 ± 0.73 | 144.34 ± 0.83 |
| SASA energy | -14.59 ± 0.02 | -25.20 ± 0.04 | -11.42 ± 0.02 | -27.36 ± 0.06 | -13.75 ± 0.02 | -23.93 ± 0.04 |
| Binding energy | -25.26 ± 0.42 | -103.93 ± 0.49 | -50.11 ± 0.86 | -128.63 ± 0.77 | -26.92 ± 0.46 | -159.90 ± 0.53 |

**Table S3:** Molecular interaction (DDS and DPC4 with Wt, Mt (T53I, P55R) obtained using Discovery Studio Visualizer.

(A) Intermolecular contacts of DDS with wild type *Ml*DHPS.

| **Interacting partners** | **Distance (Å)** | **Bond** | **Type of bonding** | | **From** | **From chemistry** | **To** | **To chemistry** |
| --- | --- | --- | --- | --- | --- | --- | --- | --- |
| **Before** | | | | | | | | |
| ARG253:HH11 - :O2 | 2.28462 | Hydrogen Bond | Conventional Hydrogen Bond | | ARG253:HH11 | H-Donor | DDS:O2 | H-Acceptor |
| ARG253:HH21 - :DDS:O3 | 2.26103 | Hydrogen Bond | Conventional Hydrogen Bond | | ARG253:HH21 | H-Donor | DDS:O3 | H-Acceptor |
| DDS:H26 -ASP86:O | 2.0902 | Hydrogen Bond | Conventional Hydrogen Bond | | DDS:H26 | H-Donor | ASP86:O | H-Acceptor |
| DDS:H27 -GLY50:O | 2.15825 | Hydrogen Bond | Conventional Hydrogen Bond | | DDS:H27 | H-Donor | GLY50:O | H-Acceptor |
| GLU51:OE2 - :DDS | 4.17579 | Electrostatic | Pi-Anion | | GLU51:OE2 | Negative | DDS | Pi-Orbitals |
| ASP86:OD2 - :DDS | 3.02118 | Electrostatic | Pi-Anion | | ASP86:OD2 | Negative | DDS | Pi-Orbitals |
| **After** | | | | | | | | |
| TYR141:HH - :DDS279:O2 | 1.58553 | Hydrogen Bond | Conventional Hydrogen Bond | TYR141:HH | | H-Donor | DDS279:O2 | H-Acceptor |
| ASP21:OD2 - :DDS279 | 3.66993 | Electrostatic | Pi-Anion | ASP21:OD2 | | Negative | DDS279 | Pi-Orbitals |
| GLU51:OE1 - :DDS279 | 4.99851 | Electrostatic | Pi-Anion | GLU51:OE1 | | Negative | DDS279 | Pi-Orbitals |
| DDS279:S1 - :TYR141 | 5.36476 | Other | Pi-Sulfur | DDS279:S1 | | Sulfur | TYR141 | Pi-Orbitals |
| DDS279- :ARG212 | 5.11439 | Hydrophobic | Pi-Alkyl | DDS279 | | Pi-Orbitals | ARG212 | Alkyl |

(B) Intermolecular contacts of DPC4 with wild type *Ml*DHPS.

| **Interacting partners** | **Distance (Å)** | **Bond** | **Type of bonding** | **From** | **From chemistry** | **To** | **To chemistry** |
| --- | --- | --- | --- | --- | --- | --- | --- |
| **Before** | | | | | | | |
| DPC4:N13 -ASP21:OD1 | 3.15481 | Hydrogen Bond | Conventional Hydrogen Bond | DPC4:N13 | H-Donor | ASP21:OD1 | H-Acceptor |
| DPC4:H2 -GLY50:O | 2.01385 | Hydrogen Bond | Conventional Hydrogen Bond | DPC4:H2 | H-Donor | GLY50:O | H-Acceptor |
| ARG214:NH2 - :DPC4 | 2.95182 | Electrostatic | Pi-Cation | ARG214:NH2 | Positive | DPC4 | Pi-Orbitals |
| ASP21:OD1 - :DPC4 | 3.26628 | Electrostatic | Pi-Anion | ASP21:OD1 | Negative | DPC4 | Pi-Orbitals |
| GLU51:OE2 - :DPC4 | 4.57461 | Electrostatic | Pi-Anion | GLU51:OE2 | Negative | DPC4 | Pi-Orbitals |
| DPC4:S20 -PHE182 | 4.16779 | Other | Pi-Sulfur | DPC4:S20 | Sulfur | PHE182 | Pi-Orbitals |
| PHE182 - :DPC4 | 4.51121 | Hydrophobic | Pi-Pi Stacked | PHE182 | Pi-Orbitals | DPC4 | Pi-Orbitals |
| TRP132 - :DPC4 | 5.22447 | Hydrophobic | Pi-Pi T-shaped | A:TRP132 | Pi-Orbitals | DPC4 | Pi-Orbitals |
| DPC4:C9 -PRO230 | 3.8841 | Hydrophobic | Alkyl | DPC4:C9 | Alkyl | PRO230 | Alkyl |
| DPC4:C9 -ARG233 | 4.54498 | Hydrophobic | Alkyl | DPC4:C9 | Alkyl | ARG233 | Alkyl |
| DPC4:C10 -ARG214 | 4.65343 | Hydrophobic | Alkyl | DPC4:C10 | Alkyl | ARG214 | Alkyl |
| DPC4:C10 -ARG233 | 4.78601 | Hydrophobic | Alkyl | DPC4:C10 | Alkyl | ARG233 | Alkyl |
| DPC4:C39 -VAL107 | 4.08322 | Hydrophobic | Alkyl | DPC4:C39 | Alkyl | VAL107 | Alkyl |
| DPC4-PRO230 | 4.99698 | Hydrophobic | Pi-Alkyl | DPC4 | Pi-Orbitals | PRO230 | Alkyl |
| **After** | | | | | | | |
| DPC4279:N13 - :GLU51:OE1 | 5.25438 | Electrostatic | Attractive Charge | DPC4279:N13 | Positive | GLU51: OE1 | Negative |
| ARG139:HH11 - :DPC4 279:O29 | 2.34664 | Hydrogen Bond | Conventional Hydrogen Bond | ARG139:HH11 | H-Donor | DPC4279: O29 | H-Acceptor |
| TYR141:H - :DPC4279:O28 | 1.67171 | Hydrogen Bond | Conventional Hydrogen Bond | TYR141:H | H-Donor | DPC4279: O28 | H-Acceptor |
| ARG139:CD - :DPC4279:O29 | 3.17394 | Hydrogen Bond | Carbon Hydrogen Bond | ARG139:CD | H-Donor | DPC4279: O29 | H-Acceptor |
| DPC4279:N12 - :PHE19 | 4.06862 | Electrostatic | Pi-Cation | DPC4279:N12 | Positive | PHE19 | Pi-Orbitals |
| DPC4279:N12 - :TYR141 | 4.26024 | Electrostatic | Pi-Cation | DPC4279:N12 | Positive | TYR141 | Pi-Orbitals |
| DPC4279:N13-:PHE19 | 4.378 | Electrostatic | Pi-Cation | DPC4279:N13 | Positive | PHE19 | Pi-Orbitals |
| DPC4279:N13-:TYR141 | 4.3309 | Electrostatic | Pi-Cation | DPC4279:N13 | Positive | TYR141 | Pi-Orbitals |
| GLU51:OE1 - :DPC4279 | 3.3075 | Electrostatic | Pi-Anion | GLU51:OE1 | Negative | DPC4279 | Pi-Orbitals |
| PHE19 - :DPC4279 | 4.2897 | Hydrophobic | Pi-Pi Stacked | PHE19 | Pi-Orbitals | DPC4279 | Pi-Orbitals |
| TYR141 - :DPC4279 | 5.75586 | Hydrophobic | Pi-Pi Stacked | TYR141 | Pi-Orbitals | DPC4279 | Pi-Orbitals |
| TYR141 - :DPC4279 | 4.70427 | Hydrophobic | Pi-Pi Stacked | TYR141 | Pi-Orbitals | DPC4279 | Pi-Orbitals |
| DPC4279:C10 - :ARG24 | 4.92527 | Hydrophobic | Alkyl | DPC4279:C10 | Alkyl | ARG24 | Alkyl |
| DPC4279:C40 - :LEU134 | 5.13173 | Hydrophobic | Alkyl | DPC4279:C40 | Alkyl | LEU134 | Alkyl |
| DPC4279:C41 - :VAL107 | 4.73153 | Hydrophobic | Alkyl | DPC4279:C41 | Alkyl | VAL107 | Alkyl |
| PHE19 -:DPC4 279:C11 | 5.08482 | Hydrophobic | Pi-Alkyl | PHE19 | Pi-Orbitals | DPC4279:C11 | Alkyl |
| TRP132 -:DPC4279:C41 | 4.64803 | Hydrophobic | Pi-Alkyl | TRP132 | Pi-Orbitals | DPC4279:C41 | Alkyl |
| TYR141 - :DPC4 279:C11 | 4.68804 | Hydrophobic | Pi-Alkyl | TYR141 | Pi-Orbitals | DPC4279:C11 | Alkyl |
| DPC4279 - :PRO140 | 3.84245 | Hydrophobic | Pi-Alkyl | DPC4279 | Pi-Orbitals | PRO140 | Alkyl |
| DPC4279 - :ALA112 | 5.26791 | Hydrophobic | Pi-Alkyl | DPC4279 | Pi-Orbitals | ALA112 | Alkyl |

(C) Intermolecular contacts of DDS with mutant type (T53I) *Ml*DHPS.

| **Interacting partners** | **Distance (Å)** | **Bond** | **Type of bonding** | **From** | **From chemistry** | **To** | **To chemistry** |
| --- | --- | --- | --- | --- | --- | --- | --- |
| **Before** | | | | | | | |
| ASN13:HD21 - DDS:O2 | 2.16633 | Hydrogen Bond | Conventional Hydrogen Bond | A:ASN13:HD21 | H-Donor | DDS:O2 | H-Acceptor |
| ARG253:HH11 - :DDS:O2 | 2.40154 | Hydrogen Bond | Conventional Hydrogen Bond | A:ARG253:HH11 | H-Donor | DDS:O2 | H-Acceptor |
| ARG253:HH21 - :DDS:O3 | 1.97203 | Hydrogen Bond | Conventional Hydrogen Bond | A:ARG253:HH21 | H-Donor | DDS:O3 | H-Acceptor |
| DDS:H26 -ASP86:O | 2.13885 | Hydrogen Bond | Conventional Hydrogen Bond | DDS0:H26 | H-Donor | ASP86:O | H-Acceptor |
| DDS:H27 -GLY50:O | 2.13969 | Hydrogen Bond | Conventional Hydrogen Bond | DDS0:H27 | H-Donor | GLY50:O | H-Acceptor |
| ASP21:OD2 - :DDS | 3.415 | Electrostatic | Pi-Anion | A:ASP21:OD2 | Negative | DDS | Pi-Orbitals |
| GLU51:OE2 - :DDS | 4.21276 | Electrostatic | Pi-Anion | A:GLU51:OE2 | Negative | DDS | Pi-Orbitals |
| ASP86:OD2 - :DDS | 2.97514 | Electrostatic | Pi-Anion | A:ASP86:OD2 | Negative | DDS | Pi-Orbitals |
| **After** | | | | | | | |
| SER20:HG - :DDS279:O3 | 2.65228 | Hydrogen Bond | Conventional Hydrogen Bond | SER20:HG | H-Donor | DDS279:O3 | H-Acceptor |
| MET130:SD - :DDS279 | 5.58858 | Other | Pi-Sulfur | MET130:SD | Sulfur | DDS279 | Pi-Orbitals |
| PHE182 - :DDS279 | 3.91762 | Hydrophobic | Pi-Pi Stacked | PHE182 | Pi-Orbitals | DDS279 | Pi-Orbitals |
| TRP132 - :DDS279 | 5.17572 | Hydrophobic | Pi-Pi T-shaped | TRP132 | Pi-Orbitals | DDS279 | Pi-Orbitals |
| TRP132 - :DDS279 | 5.47506 | Hydrophobic | Pi-Pi T-shaped | TRP132 | Pi-Orbitals | DDS279 | Pi-Orbitals |

(D) Intermolecular contacts of DPC4 with mutant type (T53I) *Ml*DHPS.

| **Interacting partners** | **Distance (Å)** | **Bond** | **Type of bonding** | **From** | **From chemistry** | **To** | **To chemistry** |
| --- | --- | --- | --- | --- | --- | --- | --- |
| **Before** | | | | | | | |
| DPC4:H1 - GLY50:O | 2.14358 | Hydrogen Bond | Conventional Hydrogen Bond | DPC4:H1 | H-Donor | GLY50:O | H-Acceptor |
| ARG214:NH2- :DPC4 | 2.72975 | Electrostatic | Pi-Cation | ARG214:NH2 | Positive | DPC4 | Pi-Orbitals |
| ASP21:OD1 - :DPC4 | 3.66547 | Electrostatic | Pi-Anion | ASP21:OD1 | Negative | DPC4 | Pi-Orbitals |
| GLU51:OE2 - :DPC4 | 4.25373 | Electrostatic | Pi-Anion | A:GLU51:OE2 | Negative | DPC4 | Pi-Orbitals |
| DPC4:S20 -PHE182 | 4.39794 | Other | Pi-Sulfur | DPC4:S20 | Sulfur | PHE182 | Pi-Orbitals |
| DPC4:C9 -:VAL107 | 4.06267 | Hydrophobic | Alkyl | DPC4:C9 | Alkyl | VAL107 | Alkyl |
| DPC4:C39 -:ARG214 | 4.38727 | Hydrophobic | Alkyl | DPC4:C39 | Alkyl | ARG214 | Alkyl |
| DPC4:C39 -:ARG233 | 5.08282 | Hydrophobic | Alkyl | DPC4:C39 | Alkyl | ARG233 | Alkyl |
| DPC4:C40 -PRO230 | 4.10206 | Hydrophobic | Alkyl | DPC4:C40 | Alkyl | PRO230 | Alkyl |
| DPC4:C40 -ARG233 | 4.62748 | Hydrophobic | Alkyl | DPC4:C40 | Alkyl | ARG233 | Alkyl |
| DPC4 -:PRO230 | 4.79701 | Hydrophobic | Pi-Alkyl | DPC4 | Pi-Orbitals | PRO230 | Alkyl |
| **After** | | | | | | | |
| DPC4279:N12 - :GLU51:OE2 | 5.05266 | Electrostatic | Attractive Charge | DPC4279:N12 | Positive | GLU51:OE2 | Negative |
| DPC4279:N13 - :GLU51:OE2 | 5.29094 | Electrostatic | Attractive Charge | DPC4279:N13 | Positive | GLU51:OE2 | Negative |
| DPC4279:N12 - :GLU51:OE2 | 5.05266 | Electrostatic | Attractive Charge | DPC4279:N12 | Positive | GLU51:OE2 | Negative |
| GLU142:H - :DPC4279:O28 | 1.80599 | Hydrogen Bond | Conventional Hydrogen Bond | GLU142:H | H-Donor | DPC4279:O28 | H-Acceptor |
| GLU142:H - :DPC4279:O28 | 1.80599 | Hydrogen Bond | Conventional Hydrogen Bond | GLU142:H | H-Donor | DPC4279:O28 | H-Acceptor |
| ALA183:H - :DPC4279:O29 | 1.73828 | Hydrogen Bond | Conventional Hydrogen Bond | ALA183:H | H-Donor | DPC4279:O29 | H-Acceptor |
| ALA183:H - :DPC4279:O29 | 1.73828 | Hydrogen Bond | Conventional Hydrogen Bond | ALA183:H | H-Donor | DPC4279:O29 | H-Acceptor |
| :DPC4279:O29 - :GLY181:O | 3.0176 | Hydrogen Bond | Conventional Hydrogen Bond | DPC4279:O29 | H-Donor | GLY181:O | H-Acceptor |
| DPC4279:O29 - :GLY181:O | 3.0176 | Hydrogen Bond | Conventional Hydrogen Bond | DPC4279:O29 | H-Donor | GLY181:O | H-Acceptor |
| TRP132:HD1 - :UNK279:O29 | 2.57404 | Hydrogen Bond | Carbon Hydrogen Bond | TRP132:HD1 | H-Donor | DPC4279:O29 | H-Acceptor |
| TRP132:HD1 - : DPC4279:O29 | 2.57404 | Hydrogen Bond | Carbon Hydrogen Bond | TRP132:HD1 | H-Donor | DPC4279:O29 | H-Acceptor |
| DPC4279:N12 - :TRP132 | 4.47898 | Electrostatic | Pi-Cation | DPC4279:N12 | Positive | TRP132 | Pi-Orbitals |
| DPC4279:N13 - :TRP132 | 4.42309 | Electrostatic | Pi-Cation | DPC4279:N13 | Positive | TRP132 | Pi-Orbitals |
| DPC4279:N12 - :TRP132 | 4.47898 | Electrostatic | Pi-Cation | DPC4279:N12 | Positive | TRP132 | Pi-Orbitals |
| VAL107 - : DPC4279 | 4.97073 | Hydrophobic | Alkyl | VAL107 | Alkyl | DPC4279 | Alkyl |
| PHE19 - : DPC4279:C19 | 5.47537 | Hydrophobic | Pi-Alkyl | PHE19 | Pi-Orbitals | DPC4279:C19 | Alkyl |
| PHE19 - : DPC4279 | 4.6657 | Hydrophobic | Pi-Alkyl | PHE19 | Pi-Orbitals | DPC4279 | Alkyl |
| PHE19 - : DPC4279:C21 | 5.3715 | Hydrophobic | Pi-Alkyl | PHE19 | Pi-Orbitals | DPC4279:C21 | Alkyl |
| TRP132 - : DPC4279:C19 | 4.60359 | Hydrophobic | Pi-Alkyl | TRP132 | Pi-Orbitals | DPC4279:C19 | Alkyl |
| TRP132 - : DPC4279:C15 | 4.20364 | Hydrophobic | Pi-Alkyl | TRP132 | Pi-Orbitals | DPC4279:C15 | Alkyl |
| TRP132 - : DPC4279 | 5.36285 | Hydrophobic | Pi-Alkyl | TRP132 | Pi-Orbitals | DPC4279 | Alkyl |
| TRP132 - : DPC4279:C19 | 5.07284 | Hydrophobic | Pi-Alkyl | TRP132 | Pi-Orbitals | DPC4279:C19 | Alkyl |
| TRP132 - : DPC4279:C11 | 5.33591 | Hydrophobic | Pi-Alkyl | TRP132 | Pi-Orbitals | DPC4279:C11 | Alkyl |
| TRP132 - : DPC4279:C15 | 4.48381 | Hydrophobic | Pi-Alkyl | TRP132 | Pi-Orbitals | DPC4279:C15 | Alkyl |
| TYR141 - : DPC4279 | 4.70896 | Hydrophobic | Pi-Alkyl | TYR141 | Pi-Orbitals | DPC4279 | Alkyl |
| PHE182 - : DPC4279:C15 | 4.67738 | Hydrophobic | Pi-Alkyl | PHE182 | Pi-Orbitals | DPC4279:C15 | Alkyl |

**(**E**)** Intermolecular contacts of DDS with mutant type (P55R) *Ml*DHPS.

| **Interacting partners** | **Distance (Å)** | **Bond** | **Type of bonding** | **From** | **From chemistry** | **To** | **To chemistry** |
| --- | --- | --- | --- | --- | --- | --- | --- |
| **Before** | | | | | | | |
| DDS:H27 -LEU12:O | 2.21257 | Hydrogen Bond | Conventional Hydrogen Bond | DDS:H27 | H-Donor | LEU12:O | H-Acceptor |
| DDS:H28 -THR87:OG1 | 2.07137 | Hydrogen Bond | Conventional Hydrogen Bond | DDS:H28 | H-Donor | THR87:OG1 | H-Acceptor |
| DDS:H29 -GLU65:OE1 | 2.10459 | Hydrogen Bond | Conventional Hydrogen Bond | DDS:H29 | H-Donor | GLU65:OE1 | H-Acceptor |
| ASP86:OD2 - :DDS | 3.20584 | Electrostatic | Pi-Anion | A:ASP86:OD2 | Negative | DDS | Pi-Orbitals |
| DDS -VAL107 | 5.14021 | Hydrophobic | Pi-Alkyl | DDS | Pi-Orbitals | VAL107 | Alkyl |
| **After** | | | | | | | |
| DDS279:H41 - :LEU12:O | 2.08485 | Hydrogen Bond | Conventional Hydrogen Bond | DDS279:H41 | H-Donor | LEU12:O | H-Acceptor |
| ASP86:OD1 - :DDS279 | 3.25047 | Electrostatic | Pi-Anion | ASP86:OD1 | Negative | DDS279 | Pi-Orbitals |
| DDS279 - :VAL11 | 5.41258 | Hydrophobic | Pi-Alkyl | DDS279 | Pi-Orbitals | VAL11 | Alkyl |
| DDS279 - :VAL107 | 4.74103 | Hydrophobic | Pi-Alkyl | DDS279 | Pi-Orbitals | VAL107 | Alkyl |

**Table S4:** Percent lethality (PL) values with mean ± standard deviation and probits during cytotoxicity of DDS and DPC4 to cultured human lymphocytes, assessed by AO/EB staining and MTT assay.

| Chemical concentration as mg/L and (Log_10_ value) | PL and probits by AO/EB staining | | PL and probits by MTT assay | |
| --- | --- | --- | --- | --- |
|  | DDS | DPC4 | DDS | DCP4 |
| 0 | - | - | - | - |
| 500 (2.69) | 2.8±0.4 (3.08) | 2.1±0.2 (2.96) | 3.2±0.4 (3.14) | 2.4±0.8(3.02) |
| 1000 (3.00) | 3.9±0.3 (3.23) | 2.9±0.1 (3.10) | 4.2±0.3 (3.27) | 3.1±0.2(3.13) |
| 1500 (3.17) | 4.8±0.3 (3.33) | 3.5±0.2 (3.18) | 6.3±1.2 (3.45) | 3.1±0.5(3.13) |
| 2000 (3.30) | 6.9±0.2 (3.51) | 4.3±0.1 (3.28) | 5.8±1.0 (3.42) | 3.5±0.1(3.18) |
| 2500 (3.39) | 10.6±0.7 (3.75) | 4.6±0.6 (3.31) | 9.1±1.9 (3.65) | 4.2±0.3(3.27) |
| 3000 (3.47) | 12.8±0.3 (3.86) | 4.9±0.9 (3.34) | 10.3±0.7 (3.73) | 5.4±0.5(3.39) |
| 3500 (3.54) | 14.4±0.7 (3.93) | 6.5±0.3 (3.48) | 13.3±0.7 (3.88) | 7.3±0.5(3.54) |
| 4000 (3.60) | 15.7±0.2 (3.99) | 6.7±1.4 (3.50) | 17.1±0.8 (4.04) | 8.8±1.0(3.64) |
| 4500 (3.65) | 15.9±1.2 (4.0) | 10.6±1.7 (3.75) | 16.8±0.9 (4.03) | 10.3±1.1(3.73) |
| 5000 (3.69) | 16.6±0.7 (4.02) | 12.7±0.7 (3.85) | 17.2±0.8 (4.05) | 11.8±0.8(3.81) |

AO/EB, Acridine orange/ethidium bromide; MTT, 3-[4, 5- dimethylthiazol-2-yl] 2,5-diphenyl tetrazolium bromide; corresponding probit values of PL are in parenthesis.

**Structural interpretation data of synthesized five DPCs.**

**DPC1**: The product was prepared by diazotized dapsone followed by coupling with 4-hydroxy coumarin as yellow crystal (yield: 85%, m.p: 316-320°C), UV-vis (λ max, ethanol): 394 nm; IR (KBr, γ, cm^-1^) : 3460 (O-H str.), 1745 (-C=O str. of lactone carbonyl), 1627 (-C=C- str. of pyrone), 1508 (-N=N- str.), 1398, 1146 (SO_2_ str. of sulfone), 1293 (C-O str.), 1103 (C-N str); ^1^H NMR (DMSO-*d_6_*, δ ppm, 400 MHz): 7.73-7.97 (m, 8H, dapsone diaryl-H), 7.89 (d, coumarinH-5), 7.45 (m, coumarinH-6), 7.69 (m, coumarinH-7), 7.52 (d, coumarinH-8); Analysis for C_26_H_18_N_4_O_6_S: calcd % C, 60.60; H, 3.05; N, 9.42;S, 5.39; Found %: C, 60.57; H, 3.01; N, 9.39; S, 5.37. MS *m/z* 595.18 (100.0 %), 596.34 (35.0 %).


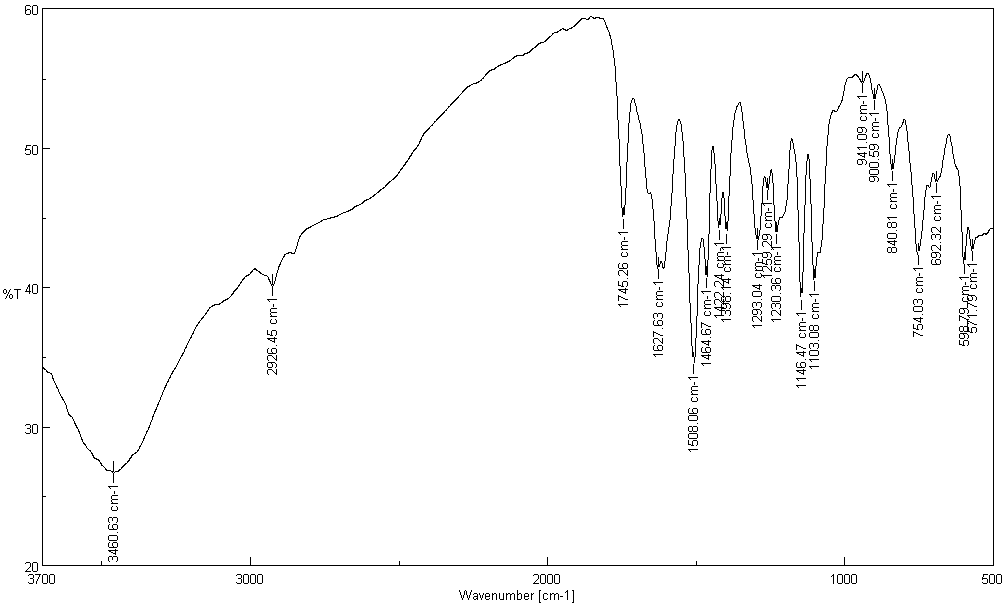


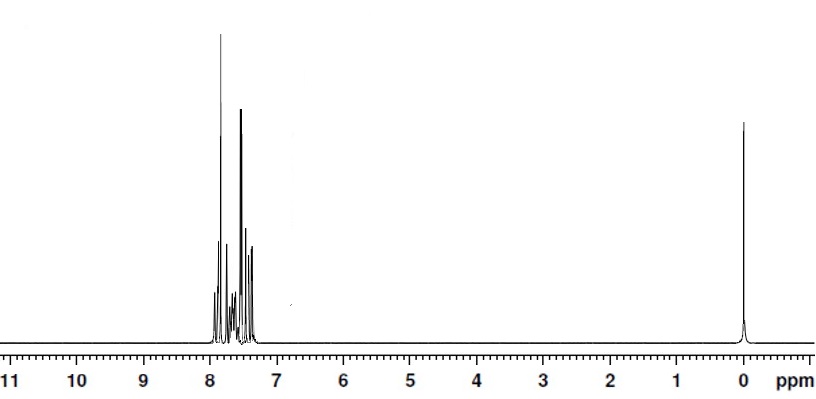


**DPC2**: The product was prepared by diazotized dapsone followed by coupling with eugenol in mild condition obtained product brown crystal (yield: 80%, m.p: 270-273°C), UV-vis (λ max, ethanol): 384 nm; IR (KBr, γ, cm^-1^): 3444 (OH str.), 3075 (C-H str.), 2931 (CH_2_ str.), 1593 (C=C str.), 1495 (-N=N- str.), 1384, 1147 (SO_2_ str. of sulfone), 1265 (-OH bend); ^1^H NMR (DMSO-*d_6_*, δ ppm, 400 MHz): 8.23-8.45 (dd, 8H, dapsone diaryl-H), 9.95 (s, 1H, OH), 3.85 (s, 3H, OCH_3_), 3.22 (m, 2H, allylCH_2_), 5.02 (d, 2H, vinylicH_a_), 4.98 (d, 2H, vinylicH_b_), 5.98 (m, 1H, vinyl H), 7.25 (s, 1H, phenylH-2), 7.07 (s, 1H, phenylH-5). Analysis for C_32_H_34_N_4_O_4_S: calcd % C, 67.35; H, 6.00; N, 9.82; S, 5.62; Found %: C, 67.32; H, 5.55; N, 9.84; S, 5.61. MS *m/z* 597.28 (100.0 %), 598.84 (35.2 %).


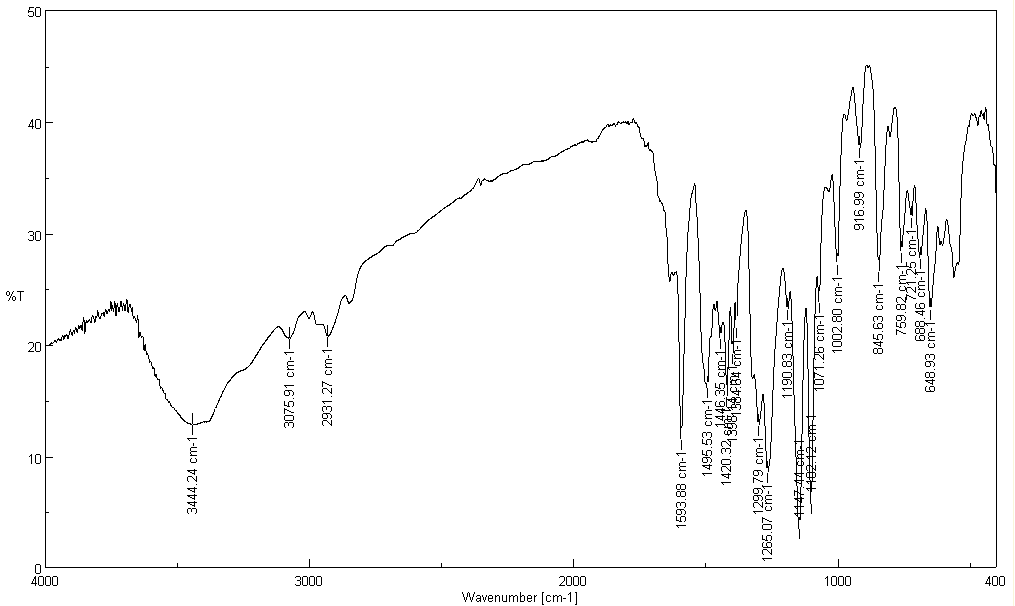


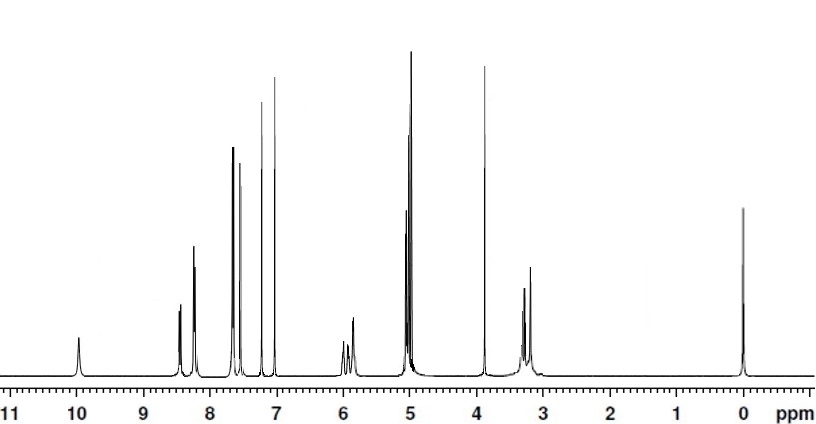


**DPC3**: The product was prepared by diazotized dapsone followed by coupling with salicylic acid as brick red crystal (yield: 78 %, m.p: 226-229°c), UV-vis (λ max, ethanol): 379 nm; IR (KBr, γ, cm^-1^) : 3373 (O-H str.), 1668 (-C=O str. of carboxylic acid), 1590 (-C=C- str. of salicylic acid), 1512 (-N=N-), 1251 (O-H bend.), 1395, 1144 (SO_2_ str. of sulfone), 1009 (C-N str.); ^1^H NMR (DMSO-*d_6_*, δ ppm, 400 MHz): 10.25 (s, 1H, COOH), 9.58 (s, 1H, OH), 8.18-8.36 (m, 8H, dapsone diaryl-H), 7.42 (dd, salicylic H-3), 7.95 (dd, salicylic H-4), 8.25 (s, 1H, salicylicH-6); Analysis for C_26_H_18_N_4_O_8_S: calcd % C, 57.14; H, 3.32; N, 10.25; S, 5.87; Found %: C, 57.11; H, 3.30; N, 10.21; S,5.86. MS *m/z* 545.10 (100.0 %), 546.24 (28.6 %).

**
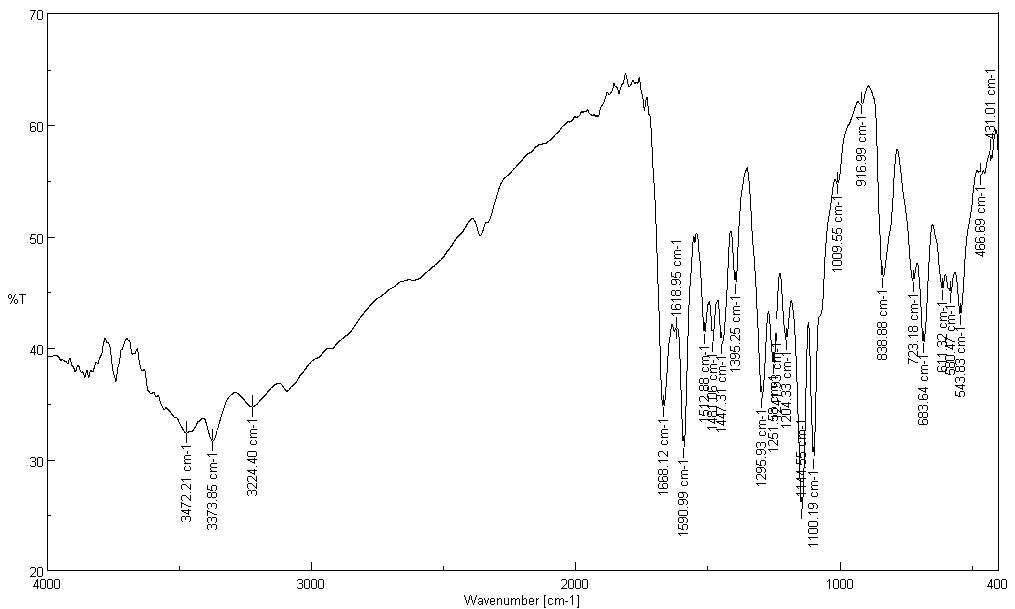
**

**
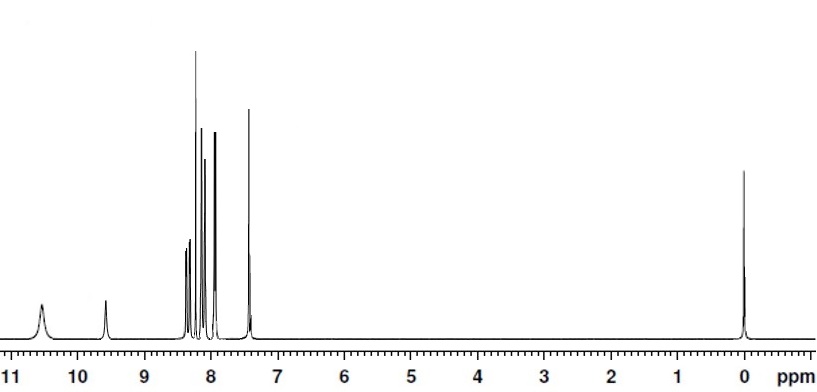
**

**DPC4**: The product was prepared by diazotized dapsone and followed by coupling with thymol as brown crystal (yield: 80 %, m.p: 272-75°c), UV-vis (λ max, ethanol): 384 nm; IR (KBr, γ, cm^-1^): 3366, 3228 (OH str.), 1631, 1590 (-C=C- str.), 1499 (-N=N-), 1278 (-OH bend), 1355, 1145 (SO_2_ str. of sulfone), 1071 (C-N str.); ^1^HNMR (DMSO-*d_6_*, δ ppm, 400 MHz): 8.17-8.39 (d, 8H, dapsone diaryl-H), 9.69 (s, 1H, Thymol OH), 2.34 (s, 3H, CH_3_ of thymol), 3.25 (s, 1H, -CH(CH_3_)_2_), 1.25 (s, 6H, -CH(CH_3_)_2_), 7.09 (s, 1H, aryl thymol H), 7.64 (s, 1H, aryl thymol H); Analysis for C_32_H_34_N_4_O_4_S: calcd % C, 67.35; H, 6.00; N, 9.82; S, 5.62; Found %: C, 67.32; H, 5.95; N, 9.84; S, 5.61; MS *m/z* 570.18 (100.0 %), 571.14 (37.0 %).


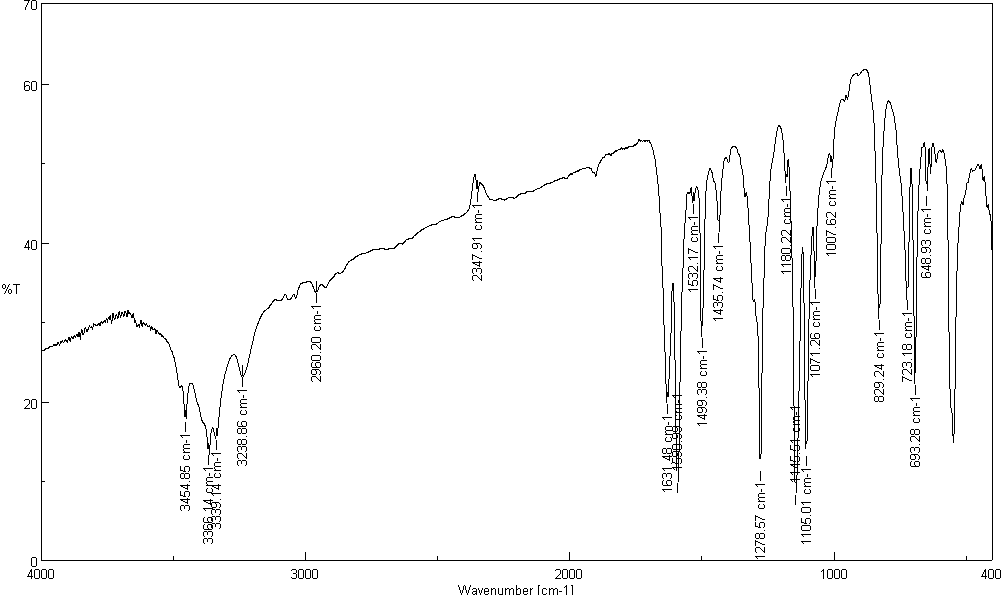


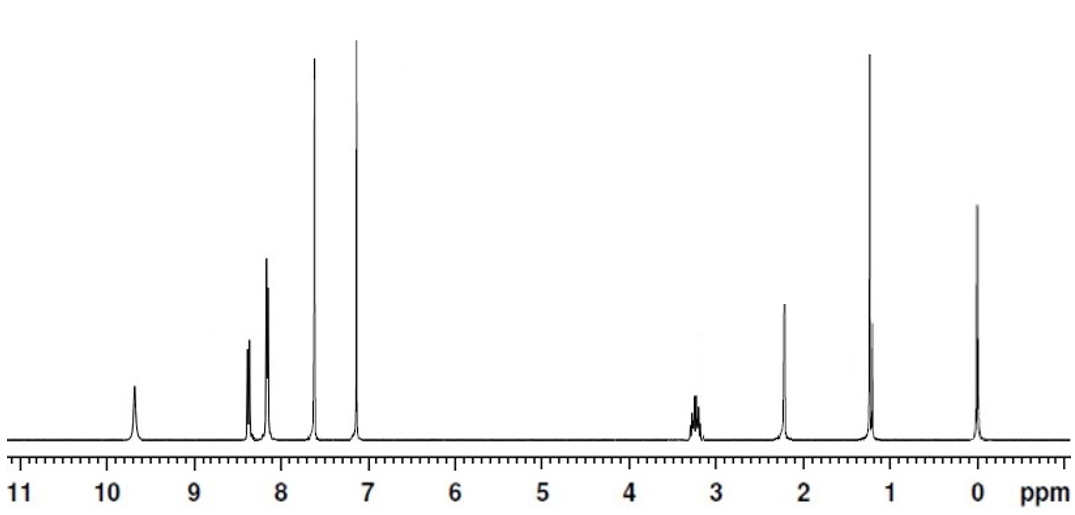


**DPC5**: The product was prepared by diazotized dapsone and followed by coupling with vanillin in mild condition obtained product brown crystal (yield: 80%, m.p: 275-77°C), UV-vis (λ max, ethanol): 384 nm; IR (KBr, γ, cm^-1^): 3391 (OH str.), 2837 (-CH_2_ str.), 1684 (C=O str. aldehyde), 1592 (-C=C- str.), 1495 (-N=N- str.), 1398, 1136 (SO_2_ str. of sulfone), 1282 (C-O str.) 1101 (C-N str.); ^1^H NMR (DMSO-*d_6_*, δ ppm, 400 MHz): 8.22-8.42 (dd, 8H, dapsone diaryl-H), 9.58 (s, 1H, CHO), 9.89 (s, 1H, OH), 7.57 (s, 1H, phenyl H-2), 7.66 (s, 1H, phenyl H-5), 3.85 (s, 3H, OCH_3_). Analysis for C_32_H_34_N_4_O_4_S: calcd % C, 67.35; H, 6.00; N, 9.82; S, 5.62; Found %: C, 67.32; H, 5.55; N, 9.84; S, 5.61. MS *m/z* MS *m/z* 573.10 (100.0 %), 574.12 (31.06 %).


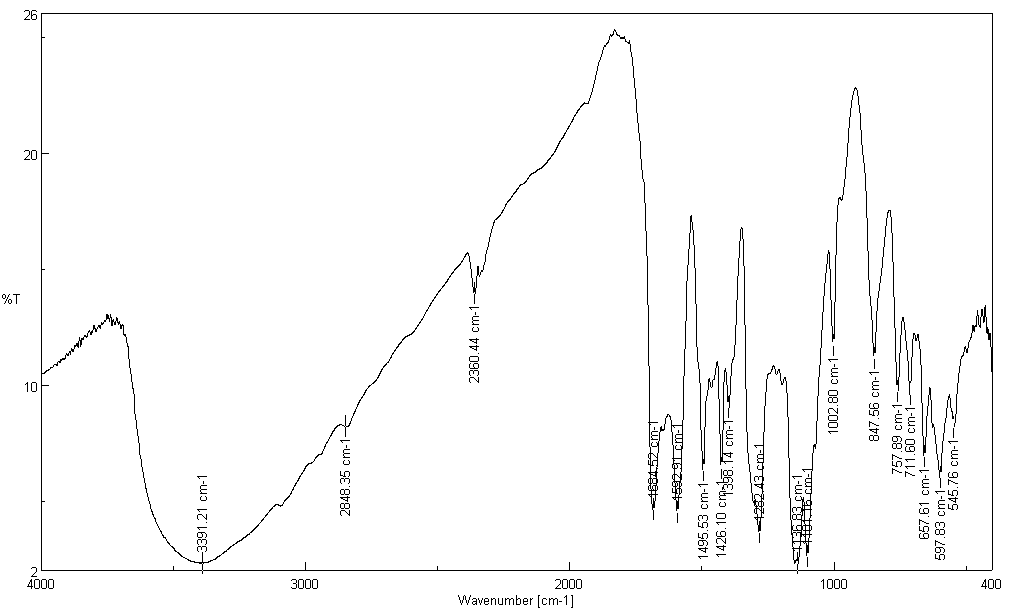


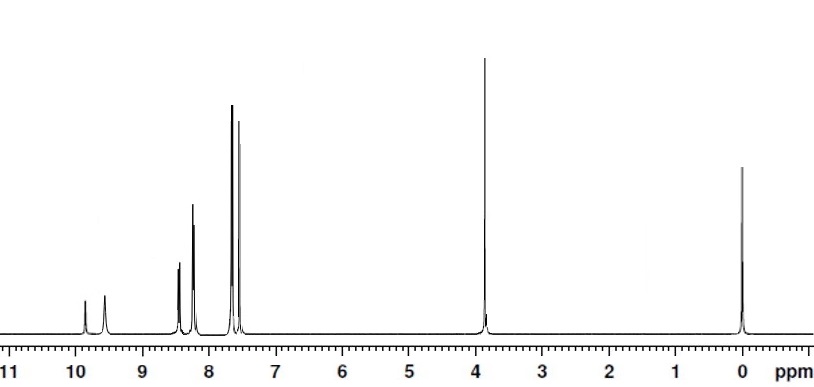

Supplement: Supplementary file 1 — Supplementary information. [file 41598_2020_63913_MOESM1_ESM.docx]
